# Photoreduction of gaseous oxidized mercury changes global atmospheric mercury speciation, transport and deposition

**DOI:** 10.1038/s41467-018-07075-3

**Published:** 2018-11-15

**Authors:** Alfonso Saiz-Lopez, Sebastian P. Sitkiewicz, Daniel Roca-Sanjuán, Josep M. Oliva-Enrich, Juan Z. Dávalos, Rafael Notario, Martin Jiskra, Yang Xu, Feiyue Wang, Colin P. Thackray, Elsie M. Sunderland, Daniel J. Jacob, Oleg Travnikov, Carlos A. Cuevas, A. Ulises Acuña, Daniel Rivero, John M. C. Plane, Douglas E. Kinnison, Jeroen E. Sonke

**Affiliations:** 1 Department of Atmospheric Chemistry and Climate, Institute of Physical Chemistry Rocasolano, CSIC, Madrid, 28006 Spain; 2 Kimika Fakultatea, Euskal Herriko Unibertsitatea UPV/EHU and Donostia International Physics Center (DIPC), P.K. 1072, Euskadi, 20080 Donostia, Spain; 3 Institut de Ciencia Molecular, Universitat de Valencia, Valencia, 46071 Spain; 4 Géosciences Environnement Toulouse, CNRS/OMP/Université de Toulouse, 31400 Toulouse, France; 5 Department of Environment and Geography, Centre for Earth Observation Science, University of Manitoba, Winnipeg, MB R3T 2N2 Canada; 6 Harvard John A. Paulson School of Engineering and Applied Sciences, Harvard University, Cambridge, MA 02138 USA; 7 Meteorological Synthesizing Centre – East of EMEP, Moscow, 115419 Russia; 8 School of Chemistry, University of Leeds, Leeds, LS2 9JT UK; 9 Atmospheric Chemistry Observations and Modelling, NCAR, Boulder, CO, 80301 USA

**Keywords:** Element cycles, Atmospheric chemistry, Environmental sciences

## Abstract

Anthropogenic mercury (Hg(0)) emissions oxidize to gaseous Hg(II) compounds, before deposition to Earth surface ecosystems. Atmospheric reduction of Hg(II) competes with deposition, thereby modifying the magnitude and pattern of Hg deposition. Global Hg models have postulated that Hg(II) reduction in the atmosphere occurs through aqueous-phase photoreduction that may take place in clouds. Here we report that experimental rainfall Hg(II) photoreduction rates are much slower than modelled rates. We compute absorption cross sections of Hg(II) compounds and show that fast gas-phase Hg(II) photolysis can dominate atmospheric mercury reduction and lead to a substantial increase in the modelled, global atmospheric Hg lifetime by a factor two. Models with Hg(II) photolysis show enhanced Hg(0) deposition to land, which may prolong recovery of aquatic ecosystems long after Hg emissions are lowered, due to the longer residence time of Hg in soils compared with the ocean. Fast Hg(II) photolysis substantially changes atmospheric Hg dynamics and requires further assessment at regional and local scales.

## Abstract (web summary)

Reduction of gaseous Hg(II) compounds drives atmospheric mercury wet and dry deposition to Earth surface ecosystems. Global Hg models assume this reduction takes place in clouds. Here the authors report a new gas-phase Hg photochemical mechanism that changes atmospheric mercury lifetime and its deposition to the surface.

## Introduction

Atmospheric mercury, a contaminant of global concern, is primarily emitted in the gaseous elemental Hg(0) form, with smaller contributions of gaseous oxidized Hg(II) and particle-bound Hg(II)^1,2^. Gaseous oxidized Hg(II)XY compounds may contain a variety of X,Y halogen atoms or oxygen-containing species, including Br, BrO, Cl, I, O, OH, HO_2_, NO_2_, and organic groups. Due to the low ambient concentration (pg m^−3^), gaseous oxidized Hg(II) compounds have only been identified as HgCl_2_ and HgBr_2_ in urban and indoor air^3^ and as HgCl_2_ in power plant plumes^4^. The atmospheric Hg(0) and Hg(II) forms have markedly different water solubility, chemical reactivity and lifetime against deposition. The lifetime of Hg(0) against deposition is in the range of several months to over a year, whereas that of Hg(II) compounds is on the order of days to weeks^5^. Eventually, Hg(0) is oxidized to Hg(II) compounds, which are soluble, partition into aerosol, and deposit readily both by dry and wet mechanisms. Direct assimilation of Hg(0) by plants and oceans is also thought to be important^6,7^. The long lifetime of Hg(0) leads to Hg deposition far from its emission sources to remote ecosystems, including the open oceans and polar regions. In aquatic ecosystems, Hg(II) is methylated and may be biomagnified up the food chain to levels that induce toxic effects in wildlife and humans^8^.

**Fig. 1:**
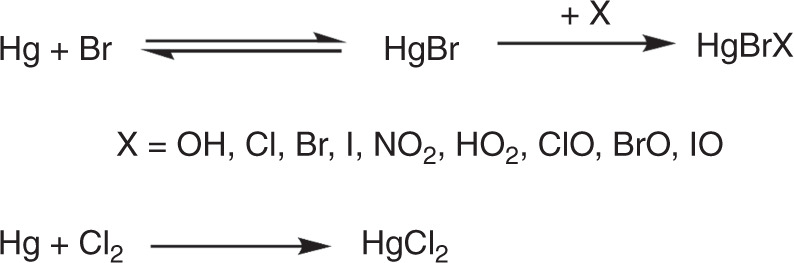
Current understanding of the formation of oxidized Hg(II) compounds from atmospheric gaseous elemental mercury initiated by different oxidant species. This figure also includes other secondary oxidation mechanisms involving single-step reactions with Cl_2_, O_3_, BrO, and ClO

The development of atmospheric chemistry and transport models (CTMs), an important tool for understanding global Hg cycling and predicting future Hg exposure, has drawn much attention to the mechanistic aspects of Hg(0) oxidation. While gas-phase O_3_, OH, HO_2_, H_2_O_2_, and NO_3_ are all potential Hg(0) oxidants^6,9–11^, the oxidation process under atmospheric conditions is thought to be initiated primarily via photolytically produced atomic bromine by a two-stage mechanism (Fig. 1)^6,11–13^. In the first step, the dominant reaction to produce gaseous oxidized Hg(II) compounds is thought to be the oxidation of Hg(0) by bromine atoms, yielding the unstable intermediate HgBr. This radical can be readily dissociated back to Hg(0), but HgBr can also be competitively oxidized by other major radical oxidant species in the atmosphere (e.g. OH, Br, I, Cl, NO_2_, HO_2_, BrO, IO, and ClO) to a series of currently-assumed stable Hg(II) compounds, as shown in Fig. 1:

Much less is known about the reduction of Hg(II) compounds to Hg(0) in the atmosphere. Global Hg CTMs, based on Hg(0) oxidation alone, predict an unrealistically short residence time of Hg(0), and the simulated spatiotemporal Hg(0) variations would not match observations^6^. To reconcile such differences, these models need to include an adjustable term to account for Hg(II) reduction in the atmosphere. Such reduction has been presumed to occur in the aqueous phase of clouds^6,10,14^. Faster gas-phase Hg(0) oxidation kinetics has led to the need of these models to employ ever faster in-cloud Hg(II) reduction^6^, with maximum rate constants > ~ 1–3 h^−1^, corresponding to in-cloud Hg(II) lifetimes <1 h on a global mean basis (see SI). Although aqueous Hg(II) photoreduction in Earth’s surface waters is a well-documented process^15^, little experimental or observational evidence exists in the case of atmospheric liquid water^16^. Earlier studies suggested that Hg(II) reduction could proceed via aqueous SO_3_ and HO_2_ reaction pathways^16,17^, but these pathways are now considered irrelevant at the global scale^6^. Therefore, the significance of atmospheric aqueous Hg(II) reduction and the validity of their inclusion in the global mercury CTMs has been questioned^6,17,18^.

None of the global mercury models has been used to test the possibility of an alternative explicit gas-phase photoreduction of Hg(II) compounds, due to the poor understanding of its mechanism and reaction rates^6^. The most recent studies—albeit 27 years ago—suggested the absence of gas-phase photoreduction for HgCl_2_ and Hg(CN)_2_ and slow photoreduction rates^19,20^ for Hg(OH)_2_ and Hg(SH)_2,_ despite an earlier study of the UV absorption cross sections which suggested that HgBr_2_ and HgI_2_ could undergo relatively fast photolysis^21,22^. As far as we are aware there have been no further experimental or theoretical studies on the photolytic properties of Hg(II) compounds of atmospheric relevance.

Here, we revisit the photoreduction pathways of atmospheric Hg(II) compounds. First, we show that irradiation experiments with boundary layer and free tropospheric rainwater do not support fast aqueous-phase Hg(II) photoreduction. We then compute the UV-VIS absorption cross sections of the following Hg(II) compounds: HgCl_2_, HgBr_2_, HgBrOCl, HgBrI, HgBrOBr, HgBrOI, HgBrNO_2_, HgBrONO, HgBrOH, HgBrOOH, and HgO, using high-level quantum chemical methods, and infer the corresponding atmospheric photoreduction rates. Our results show for the first time that gas-phase Hg(II) photoreduction can proceed at relevant timescales, and is more important than in-cloud Hg(II) photoreduction. The inclusion of this new gaseous-phase Hg(II) photoreduction mechanism in two state-of-the-art global Hg models reveals major implications for our understanding of Hg cycling in the atmosphere, and its deposition to the surface environment.

## Results

### Laboratory rainfall Hg(II) photoreduction experiments

To study aqueous phase Hg(II) photoreduction, ten rainfall events were sampled in suburban Toulouse and at the high altitude (2877 m) Pic du Midi Observatory (PDM, France) in the summer of 2017. Rainfall samples were irradiated in a quartz reactor with natural sunlight or with a solar simulator (see Methods). We observe (Supplementary Figure 1a and 1b, Supplementary Data 1 and Supplementary Table 1) no statistically significant differences between rainfall Hg(II) reduction rates under natural (0.063 ± 0.013 h^−1^) and simulated sunlight (0.037 ± 0.016 h^−1^), and for filtered (0.058 ± 0.011 h^−1^) and unfiltered (0.039 ± 0.020 h^−1^) suburban rainwater (*t*-test, all *p* > 0.05). The mean photochemical reduction rate of suburban rainfall was 0.051 ± 0.019 h^−1^ (σ, *n* = 10). The mean rate at the remote PDM samples was two-fold higher, 0.15 ± 0.01 h^−1^ (σ, *n* = 3), than that of the suburban Toulouse samples, and three times slower than the median photoreduction rate of 0.41 h^−1^ (*n* = 24) for inland and marine waters^15^. Our experimental rainwater photoreduction rates, under fully sunlit conditions, are an order of magnitude slower than the optimized maximum in-cloud photoreduction rate^6,23^ of > 1.0 h^−1^ in global Hg CTMs.

### Quantum chemical computation of gaseous Hg(II) absorption cross sections

**Fig. 2:**
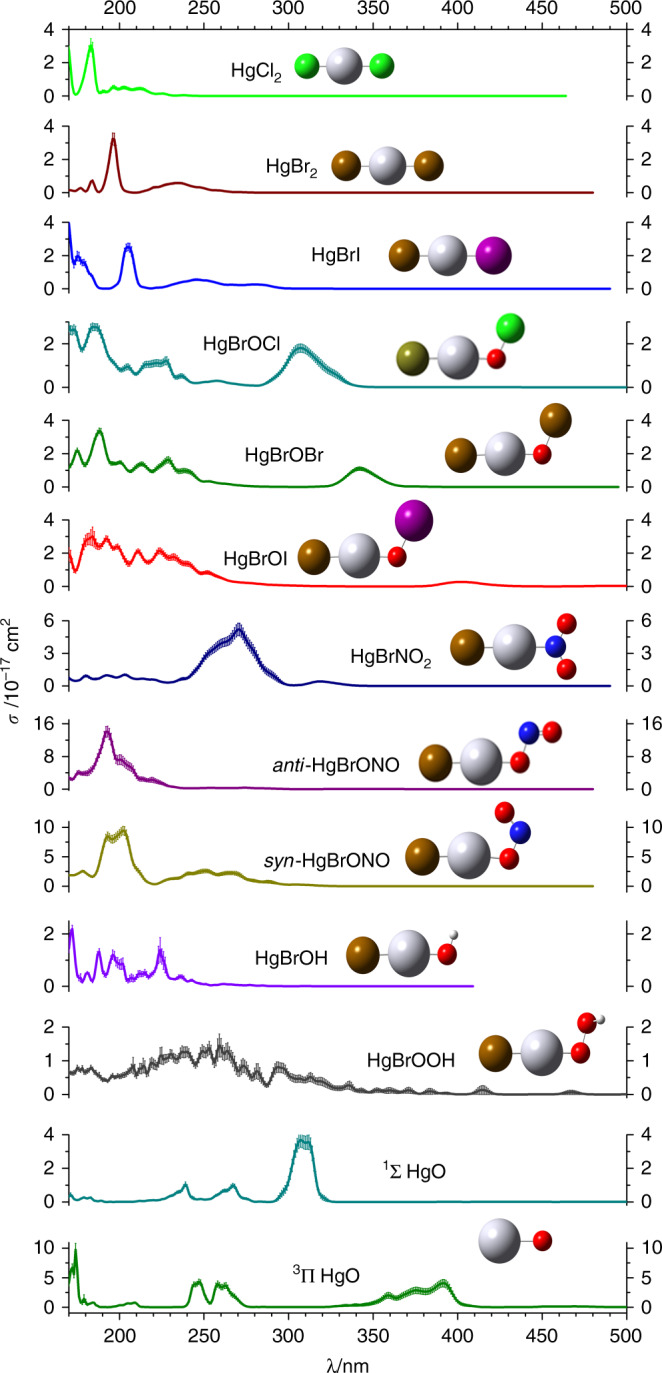
Ball-and-stick representation and computed UV-VIS absorption spectra and cross sections (σ, cm^2^) of the Hg(II) compounds studied in the present work. The light-coloured areas correspond to the uncertainty of the cross section due to the statistical sampling. Note the different range of σ values for some of the spectra. Also note that only wavelengths >290 nm are relevant for ambient tropospheric conditions

**Fig. 3:**
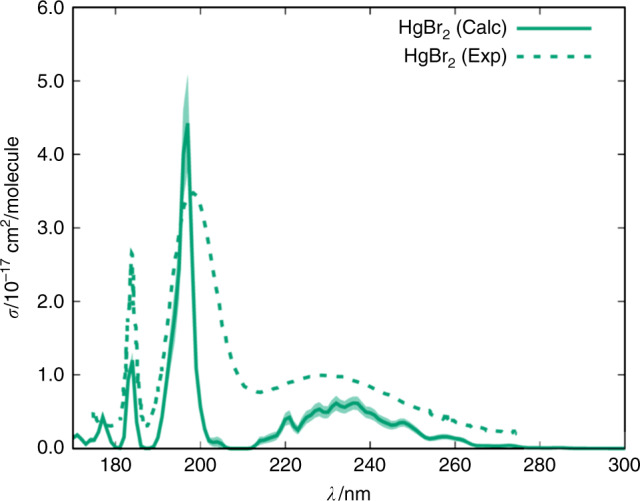
Calculated and experimental cross section of gas-phase HgBr_2_. The calculated spectrum was obtained with the CASSCF/MS–CASPT2/SO RASSI methodological approach using the ANO-RCC-VTZP basis set. The light coloured areas correspond to the numerical error of absorption cross sections due to the statistical sampling

We now turn to the computation of electronic spectra and absorption cross sections of gas-phase Hg(II) compounds which are required to estimate the corresponding photoreduction rates. A summary of the UV-VIS spectra and absorption cross sections, computed at the CASSCF/MS–CASPT2/SO–RASSI level of theory (Methods), is presented in Fig. 2 for the 170–600 nm wavelength range. The calculated spectra of HgCl_2_ and HgBr_2_ are in very good agreement with previous experimental^22,24–26^ and computed spectra^21^ (Fig. 3), thus providing strong support for the theoretical method applied here. The majority of the spectra consist of well-defined absorption bands in the 200–350 nm range, which are red-shifted when Cl is replaced with Br and I atoms. Note that three different isomers could form from the reaction of HgBr with NO_2_: HgBrNO_2_, and *syn*- and *anti*-HgBrONO. However, high-level quantum chemical computations^27^ indicate that *syn*-HgBrONO is the most thermodynamically stable species.

### Computation of photolysis rates and atmospheric lifetime of Hg(II) compounds

**Fig. 4:**
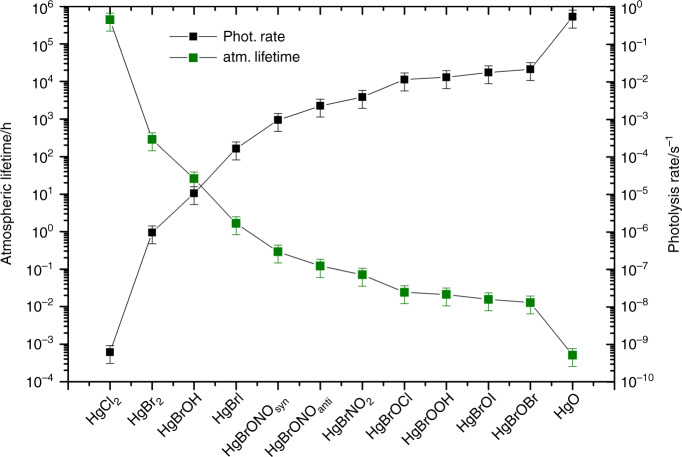
Annually- and globally-averaged photolysis rate (s^−1^) and lifetime (h) of Hg(II) compounds in the troposphere. Error bars correspond to one standard deviation

The annually averaged atmospheric lifetimes against photolysis in the troposphere for the Hg(II) compounds studied here are presented in Fig. 4 (see also Supplementary Table 2 and Supplementary Figure 2 for zonal-averaged atmospheric lifetimes). The species with the longest lifetime is HgCl_2_ (48 years), and the species with the shortest lifetime is HgBrOBr (<1 s). These lifetimes were calculated assuming a complete UV-VIS photodissociation under atmospheric conditions. In the case of the parent HgBr_2_ compound, it is well known that irradiation with ~ 200 nm UV light yields the monohalide (HgBr) with nearly 100% efficiency^25,28–30^. Moreover, detailed quantum-chemical computations of the Cl- and Br-dihalides predict further efficient photodissociation at wavelengths^21^ longer than 200 nm. There are no comparable experimental or calculated photolysis data for the other HgBr-X compounds studied here. Nevertheless, a similar very efficient photodissociation step is to be expected for these mercury halides considering the even lower dissociation energies of the HgBr-X bond, as compared with that of the parent HgBr-Br dihalide (Supplementary Table 3)^9,27^. In addition to this primary photolysis reaction to HgBr, it has been shown that Hg(0) is also generated in the HgBr_2_ photodissociation through direct or secondary channels, although to a much lesser extent^31,32^. Based on this evidence, we consider in the atmospheric modelling below that HgBr is the main product of HgBrX photodissociation, although we also ran one scenario where HgBrX photodissociation results in Hg(0) production.

## Discussion

**Fig. 5:**
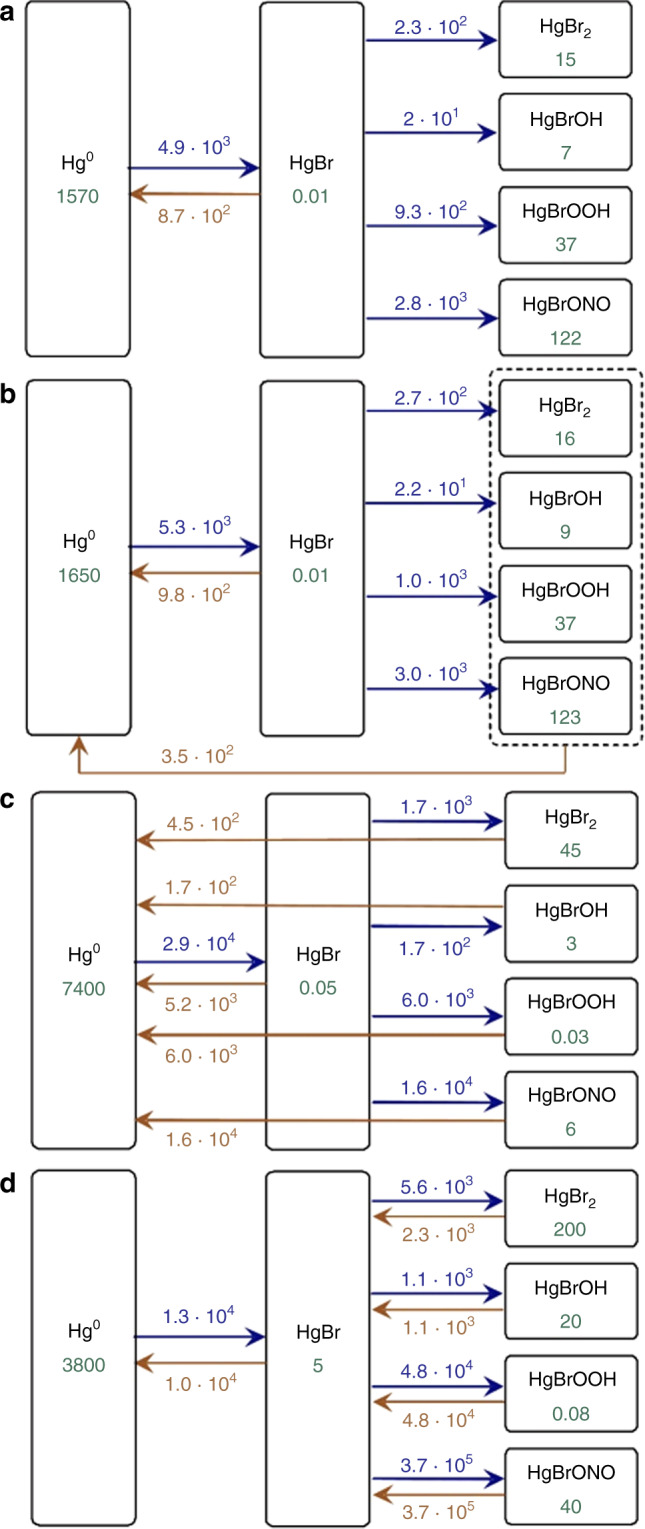
Global budget of Hg chemical cycling covering the troposphere and lower stratosphere (up to ca. 30 kms) for different tests in GLEMOS: **a**—Run #1; **b**—Run #2; **c**—Run #3; **d**—Run #4. The mass estimates are in Mg, the fluxes are in Mg a^−1^

The absorption cross sections of *syn*-HgBrONO, HgBrOOH, HgBrOH, HgBr_2_, HgBrOCl, and HgBrOBr were implemented into the GEOS-Chem^6^ and GLEMOS^33,34^ global Hg chemistry and transport models (Methods), since these Hg(II) species are the most likely to be formed in the atmosphere^6,11,27^. GEOS-Chem simulates Hg(II) as a single tracer, whereas GLEMOS simulates Hg(II) species individually. In GEOS-Chem the rapidly photolyzing Hg(II) species (HgBr-[ONO, OOH, OCl, OBr]) are calculated to be at pseudo-steady-state with HgBr. GLEMOS does not include the highly uncertain reduction reaction^6^ HgBr + NO_2_ → Hg(0) + BrNO_2_. Omitting this reaction in GEOS-Chem lowers the Hg lifetime from 13 to 8 months in model Run#4 (The different model simulated scenarios for atmospheric Hg(II) reduction are shown in Table 1). *syn*-HgBrONO and HgBrOOH generally dominate the production of Hg(II) in both models (Supplementary Figure 3), whilst HgBr_2_ becomes the prevalent Hg(II) species in the troposphere (Supplementary Figure 4) due to its longer lifetime against photolysis. Note that direct photoreduction to Hg(0) produces unrealistically long Hg lifetimes >19 months in both models. Therefore, photoreduction was considered to produce HgBr in all cases. Indeed, intensive photolysis of *syn*-HgBrONO and HgBrOOH causes HgBr to be a relevant species in the free troposphere (Supplementary Figure 5 and Fig. 5). HgBr can then be re-oxidized to gaseous Hg(II), or decay to Hg(0) by thermal dissociation, which is strongly dependent on pressure and temperature^6,9^. Atmospheric aqueous Hg(II) reduction parameterizations in both models were capped with an upper limit that corresponds to our observed rainfall photoreduction rate constant, *k*_red_ = 0.15 (h^−1^), leading to a 6% decrease in modelled atmospheric lifetime. Published model runs with capped aqueous phase Hg(II) reduction and without the gas-phase photoreduction^6,33,34^ yield total atmospheric Hg lifetimes of 4.9 and 4.6 months against deposition. Our new results show that gaseous Hg(II) photoreduction increases the Hg lifetime to 13 and 10 months in GEOS-Chem and GLEMOS model Run#4, respectively. We find that gas-phase photoreduction is the dominant reduction pathway.

**Table 1:** Model test runs for different atmospheric Hg(II) reduction scenarios in the GLEMOS model

Run ID	Scenario
Run #1	No Hg(II) reduction
Run #2	Hg(II) reduction in aqueous phase using the experimentally derived rate constant (0.15 h^−1^) in this study
Run #3	Gas phase Hg(II) photoreduction to Hg(0)
Run #4	Gas phase Hg(II) photoreduction to HgBr

**Fig. 6:**
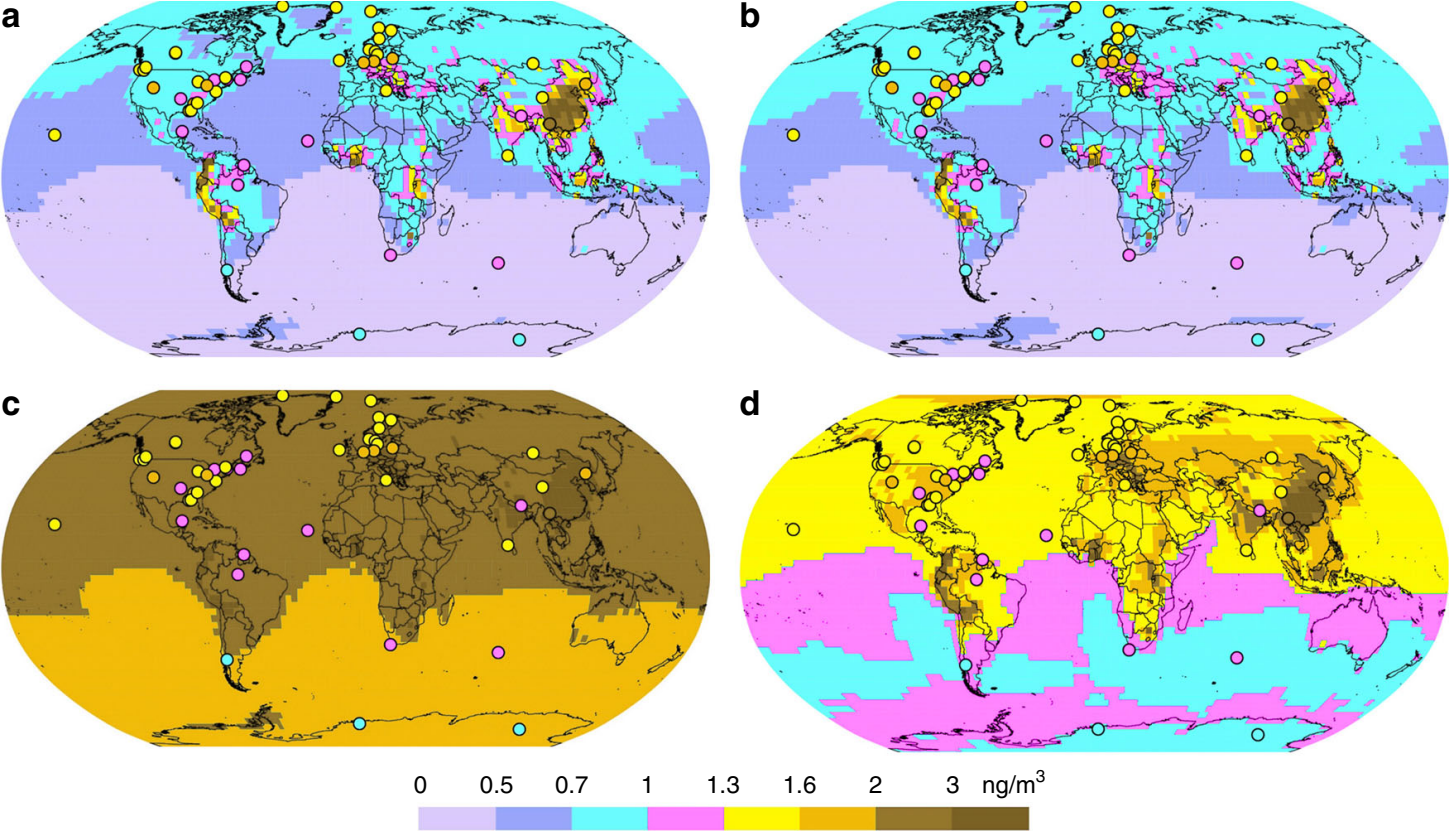
Spatial distribution of Hg(0) surface concentration for different atmospheric Hg(II) reduction simulations in the GLEMOS model: **a** Run #1; **b** Run #2; **c** Run #3; **d** Run #4. Circles show observed values in the same colour scale. The measurement dataset is the same as in ref. ^35^

**Fig. 7:**
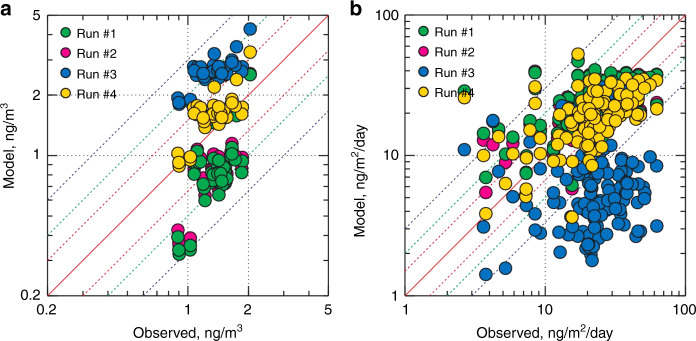
Comparison between simulations from the GLEMOS model with measurements for the year 2013: **a** Hg(0) air concentration; **b** Hg(II) wet deposition. The measurement dataset is the same as in ref. ^35^

**Fig. 8:**
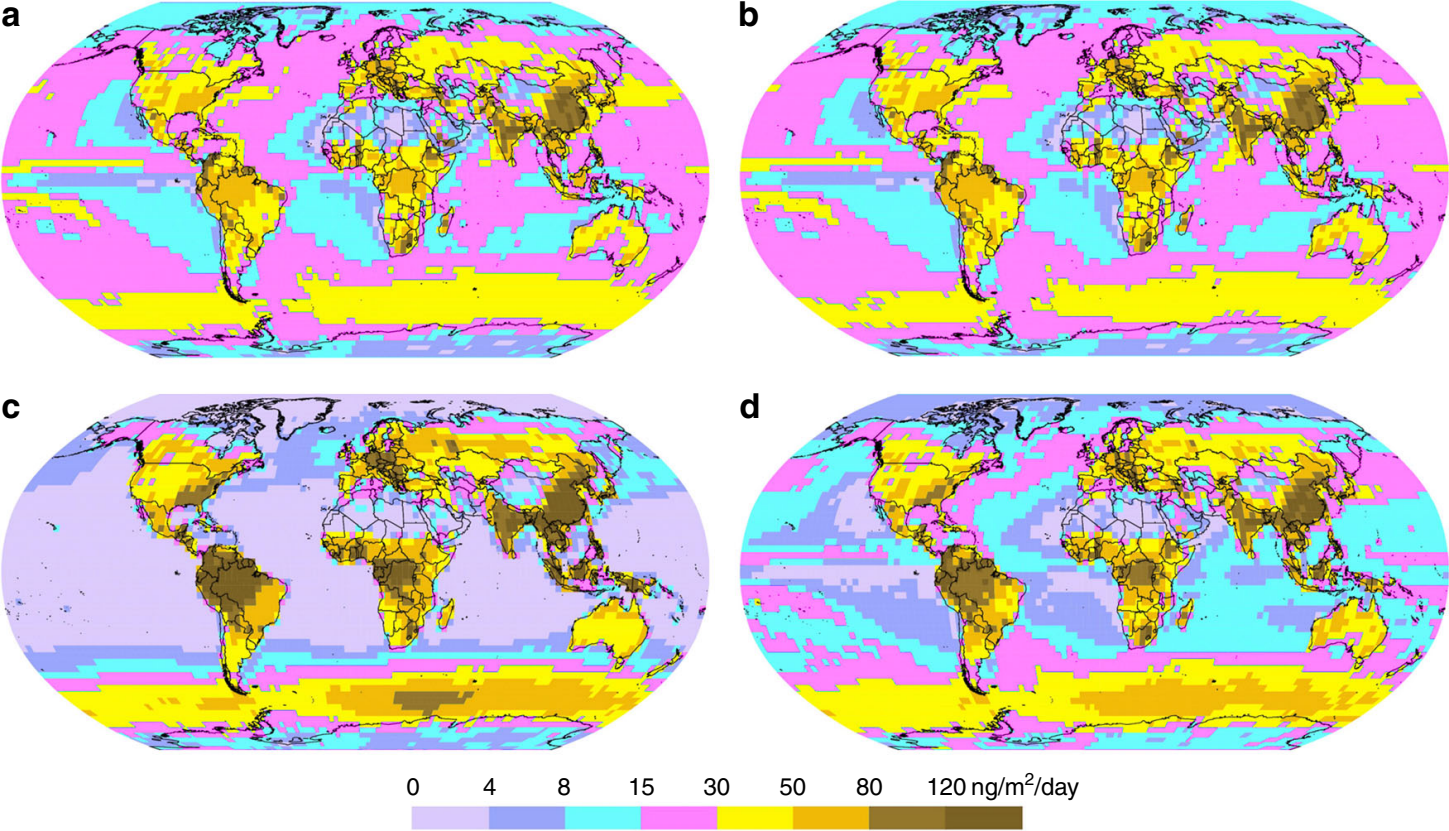
Spatial distribution of total Hg (i.e. Hg(0) + Hg(II)) deposition for different tests in GLEMOS: **a**—Run #1; **b**—Run #2; **c**—Run #3; **d**—Run #4

We further examined the global atmospheric Hg(0) and Hg(II) distribution in GLEMOS. Figures 6 and 7 show the effect of the new photoreduction scheme on the global distribution of Hg(0) surface concentration. All these simulations were made with the previously assumed aqueous photoreduction mechanism removed. We find that simulations without the gaseous photoreduction lead to 35−40% underestimation of observed Hg(0) (Runs #1 and #2). The gas-phase Hg(II) photoreduction to Hg(0) (Run #3) results in unrealistically high Hg(0) concentrations with almost two-fold overestimation of the observations and strong underestimation of wet deposition. The model run with the incorporation of the gas phase photoreduction to HgBr (Run #4) shows that Hg(0) levels are 18% overestimated, and model Hg(II) wet deposition 20% underestimated with respect to observations (Fig. 7, Supplementary Table 4). The results of test Run#4 are closest to the observations which suggest that gas-phase reduction processes are important but also that re-oxidation via the HgBr intermediate is important. An additional step in the evaluation of model results can be made by examining the variability of modelled and observed Hg concentrations. Previous studies indicated that longer Hg(0) lifetimes lead to lower simulated Hg(0) variability, as represented by the standard deviation (1σ) of mean Hg(0) concentrations^6,34^. Here, gas-phase photoreduction leads to simulated Hg(0) levels at the measurement sites (1.62 ± 0.36 ng/m^3^, 1σ, STP) that have a larger standard deviation than observed Hg(0) (1.38 ± 0.25 ng/m^3^, 1σ, STP). This indicates that the longer Hg(0) lifetime estimates of 8–13 months resulting from model Run#4 are broadly compatible with observed Hg(0) variability (1σ). The resulting global zonal distribution of Hg(0) and speciated Hg(II) (*syn*-HgBrONO, HgBrOOH, HgBrOH, HgBr, HgBr_2_) reveals the major effects of gas-phase Hg(II) photoreduction in the global budget of atmospheric oxidized mercury (Supplementary Figures 4–9) and in the global patterns of mercury surface deposition (Fig. 8 and Supplementary Figure 10). In particular, it leads to a strong decrease of free tropospheric concentrations of *syn-*HgBrONO and HgBrOOH, which were previously considered as dominant Hg(II) species. In contrast, concentrations of HgBrOH and HgBr_2_ increase due to the lower photolysis rates. The incorporation of gas-phase photoreduction leads to an increase in global Hg(0) deposition from 11% (Run#1) to 24% (Run#4) at the expense of Hg(II) deposition (down by 13%, Fig. 8 and Supplementary Figure 10). We further observe a reduction of Hg deposition (dry and wet) to the ocean (down by 15%, Fig. 8 and Supplementary Figure 10), and an increase of Hg(0) dry deposition to the land surface (22%), particularly to vegetation in line with the recent findings that foliar uptake by vegetation drives continental Hg(0) seasonality^7^. Global chemical budget diagrams (Fig. 5) summarize the Hg(0), Hg(I) and Hg(II) cycling in different model runs.

This work shows that the presence of an efficient gas-phase Hg(II) photoreduction challenges the current understanding of Hg cycling in the atmosphere and its deposition to the surface environment. We show that the new gas-phase Hg(II) photoreduction mechanism is likely the dominant reduction pathway for atmospheric mercury which can change the concept of the speciation of Hg(II) in the atmosphere. Its inclusion in state-of-the-art global models leads to significant modifications in the local scale deposition of Hg to the Earth’s surface. As a result, enhanced deposition to land surfaces may prolong recovery of aquatic ecosystems long after Hg emissions are curbed, due to the longer residence time of Hg in soils than in oceans^35^.

## Methods

### Computation of UV-Vis absorption spectra and cross sections

A theoretical methodology previously calibrated^36^ was used for calculating the electronic absorption spectra and cross-sections of HgCl_2_, HgBr_2_, HgBrI, HgBrOBr, HgBrOI, HgBrOCl, HgBrNO_2_, HgBrONO, HgBrOH, HgBrOOH, and HgO (The absorption cross sections are reported in Supplementary Data 2). Specifically, vertical transitions energies from the ground to electronically excited states and the corresponding oscillator strengths were computed with the highly accurate multireference complete-active-space self-consistent field/multistate complete-active-space second-order perturbation theory (CASSCF/MS-CASPT2) method^37^, and the atomic-natural-orbital relativistic correlation-consistent valence triple-ζ plus polarization (the ANO-RCC-VTZP) basis set^38^, taking into account scalar relativistic and spin-orbit coupling (SOC) effects (see below and Supplementary Table 5 for further details). Scalar relativistic effects were included by means of the third-order Douglas-Kroll and Hess (DKH3) Hamiltonian, and the spin-orbit coupling (SOC) was computed using the restricted active space state interaction (RASSI) method, as implemented in the MOLCAS 8 program^39^.

The atmospheric modelling methods used in the present work require as input data the absorption cross sections of any compound that may undergo photolysis in the UV-VIS range under atmospheric conditions. To estimate the vibrational resolution and determine the band shapes, absorption spectra were generated for all the compounds studied here by sampling the nuclear coordinates of the ground-state equilibrium structure and frequencies according to a Wigner distribution, as described in refs ^40,41^ and subsequently computing the vertical transition energies and oscillator strengths at each structure. The Wigner distribution of geometries was obtained with the Newton-X 1.4 program^42,43^, and an in-house program was used to compute the cross sections from the energies and oscillator strengths generated by the MOLCAS program. The ground-state structures and frequencies needed to generate the Wigner distribution were obtained by using the PBE0 functional^44^ with the Def2QZVP basis set^45–47^ as implemented in the Gaussian 09 package^48^. The minor differences observed in the simulated spectra due to ground-state geometries generated using either CASPT2, CCSD, or DFT methods are presented in Supplementary Figure 11.

For the di- and triatomic systems, appropriate symmetry point groups available in the MOLCAS program and which enabled for every possible displacement of atoms, were used in the calculations—C_2v_ in HgO and C_s_ in HgCl_2_, HgBr_2_, and HgBrI. For other studied systems, no symmetry was adopted (C_1_ group). For each particular system, the number of spin-free states to account for in the CASSCF/MS-CASPT2 calculations were selected according to the energy criteria in such way to include all relevant electronic transitions up to 170 nm (and later apply SOC effects). All the parameters of the carried simulations of the spectra, such as the number of sampled geometries N_p_, broadening of the Gaussian shape functions δ, and the numbers of included states per symmetry, are presented in detail in Supplementary Table 5.

By using the Wigner distribution of geometries for estimating the vibrational structure of the spectra and the CASSCF/MS-CASPT2/SO-RASSI methodology for determining the electronic structure, the agreement between computed and experimental transition energies was in the range of 5-10%, for those few cases in which gas-phase experimental data were available, namely for mercury compounds HgCl_2_ and HgBr_2_ (Fig. 3). The corresponding uncertainty in the calculated cross-section values is ± 25% for the most intense transitions (see Fig. 3), similar to the actual dispersion of the experimental values^22,24–26^.

The selected active spaces for the CASSCF/MS-CASPT2 computations are briefly discussed here. First general details are given and next we discuss those aspects which refer to each group of compounds.

According to our previous work on benchmarking the methodology for the representative HgBr_2_ molecule^36^ and on the basis of several test CASSCF computations with distinct active spaces for the whole set of molecules, some rules regarding the selection of the active spaces could be established.

First, for all the systems, s-subshell orbitals (and electrons) are not relevant in the studied energy range (up to 170 nm) and therefore were kept inactive and doubly occupied, except for the 6s orbital of Hg, which has a key role in the transitions.

Second, the 5d orbitals of Hg are not involved in the transitions within the energy region of our interest, and therefore were not correlated in the CASSCF/MS-CASPT2 simulations for any system with the exception of the 1^1^Σ and 1^3^Π states of HgO. For this system, tests showed a small contribution of the 5d orbitals for high-energy transitions close to 170 nm.

Third, the 6p orbitals of Hg, especially those perpendicular to σ orbitals of Hg-X, namely 6p_x_ and 6p_y_, should be correlated at the CASSCF level. On the other hand, test calculations have shown that the Hg atomic orbital of 6p_z_-type, colinear with mercury covalent bonds, has no weight in the transitions relevant for this study, and was not included in the active spaces for the larger-size systems.

Finally, the two last natural orbitals (NOs) of non-bonding character, consisting on Br 4d_xz/yz_ + Br 4d_xz/yz_ atomic orbitals (AOs) and used in our first benchmark study on HgBr_2_^36^, were not necessary for the computations and were omitted in the di- and triatomic systems, in order to reduce computational effort.

For each particular compound, the following criteria were adopted:

For HgCl_2_ HgBr_2_ and HgBrI, the optimal active spaces consisted of 12 electrons distributed in 10 NOs of the following character: σ/σ^*^-type(Hg 6s ± Cl 3p_z_/ Br 4p_z_/ I 5p_z_ and Hg 6p_z_ ± Cl 3p_z_/Br 4p_z_/I 5p_z_), σ^nb^-type (Cl 3p_z_/Br 4p_z_/I 5p_z_), and π^nb^-type (Cl 3p_x/y_/Br 4p_x/y_/I 5p_x/y_ and Hg 6p_x/y_). Although included in the active space, the σ^*^ orbital, Hg 6p_z_ ± Cl 3p_z_ / Br 4p_z_ / I 5p_z_ was not significantly occupied in the electronic configurations.

For ^1^Σ HgO and ^3^Π HgO the optimal active spaces consisted of 16 electrons distributed in 12 NOs. For these systems only, the active space additionally had to include 10 electrons belonging to the Hg 5d-subshell, and the remaining NOs were of the following type: σ/σ^*^-type (Hg 6s ± O 2p_z_ and Hg 6p_z_ ± O 2p_z_), and π^nb^-type (O 2p_x/y_ and Hg 6p_x/y_). In contrast to the other systems studied, σ^*^ Hg 6p_z_ + O 2p_z_ had an observable contribution in the configuration characterizing the excited states.

For HgBrOBr and HgBrOI the optimal active spaces consisted of 16 electrons distributed over 12 NOs. They correspond to the NOs formed by Hg 6s and AOs of p-type: O 2p_x/y/z_, Br 4p_x/y/z_/I 5p_x/y/z_ and Hg 6p_x/y_. Tests have shown that for the energy region of interest in the UV-VIS range, there are no relevant transitions to the Hg 6p_z_ orbital, and therefore it was not included in the active space to speed up the calculations.

For *syn*-HgBrONO and *anti*-HgBrONO and HgBrNO_2_. isomers, the optimal active spaces consisted of 16 electrons distributed in 12 NOs. They correspond to NOs formed by Hg 6 s and the AOs of p-type: O 2p_x/y/z_, Br 4p_x/y/_ and Hg 6p_x/y_. Some orbitals from the valence space of the ONO and NO_2_ groups had to be omitted due to the computational limitations. Tests have shown that for the relevant wavelength region in the UV-VIS, one σ-type bonding NO at the O-N-O and NO_2_ groups of atoms remains doubly occupied and it was moved to the inactive space, whereas the Hg 6p_z_ orbital and higher σ^*^-type virtual orbitals do not contribute significantly and therefore were kept in the secondary space.

In the case of HgBrOOH, the optimal active space was similar to those of HgBrOBr and HgBrOI. It consisted of 16 electrons distributed in 12 NOs formed by Hg 6 s and AOs of p-type: O 2p_x/y/z_, Br 4p_x/y/_ and Hg 6p_x/y_. As for in previous cases, the Hg 6p_z_ orbital was kept inactive since it was not relevant. In the case of HgBrOH, the appropriate active space was 12 electron distributed in 11 NOs.

To test the effect of the geometries used in the Wigner distribution on the simulated UV-VIS spectra, the optimization of the geometry of the ground state and the frequencies of normal modes were obtained using three different quantum-chemical methods for the representative HgBr_2_ molecule:

- DFT/PBE0/Def2QZVP,
- CCSD/Def2QZVP, and
- SS-CASPT2(12,10)/ANO-RCC-VTZP.

Next, the computations of the electronic structure of the excited states were done for each set of geometries at the same level of theory: SOC-DKH3-MS-CASPT2(12,10)/ANO-RCC-VTZP. The same number of sampled geometries *N*_p_ and broadening of the Gaussian shape functions *δ*, were also chosen for the three sets of calculations: *N*_p_ = 100 and *δ* = 0.05 eV, as shown in Supplementary Table 5.

The UV-VIS absorption spectra obtained when using the differently generated sets of geometries is presented in Supplementary Figure 11 for HgBr_2_. As can be seen, no significant differences are obtained, which validates the use of the less computationally costly method (DFT) for the simulations in the other molecules.

### Computation of the photolysis rates

In this study we employ the global 3D chemistry-climate model CAM-Chem (Community Atmospheric Model with chemistry, version 4.0), to estimate the photolysis rate (*J*), and therefore the atmospheric lifetime (*τ* = 1/*J*), of the different Hg(II) species according to their computed absorption cross section. The model includes a comprehensive chemistry scheme to simulate the evolution of trace gases and aerosols in the troposphere and the stratosphere^49^. The model runs with the chlorine, iodine and bromine chemistry schemes from previous studies^50–52^, including the photochemical breakdown of bromo- and iodo-carbons emitted from the oceans^49^ and abiotic oceanic sources^53^ of HOI and I_2_. We have included all the Hg(II) species (HgCl_2_, HgBr_2_, HgBrI, HgBrOCl, HgBrOBr, HgBrOI, HgBrNO_2_, HgBrONO (*syn* and *anti*), HgBrOH, HgBrOOH, and HgO) and their computed absorption cross sections. CAM-Chem has been configured in this work with a horizontal resolution of 1.9° latitude by 2.5° longitude and 26 vertical levels, from the surface to ∼ 40 km altitude. The model run in this study was performed in the specified dynamics mode^49^ using offline meteorological fields instead of an online calculation. This offline meteorology consists of a high-frequency meteorological input from a previous free running climatic simulation^54^.

### Description of the GEOS-Chem model

In this study, we use the GEOS-Chem Hg simulation from ref. ^6^ using the surface slab ocean boundary parametrization^55^. The model calculates the transport and chemistry of tracer species Hg(0) and Hg(II). The parametrization of gas-particle partitioning of Hg(II) is from ref. ^56^, and the mercury redox chemistry (described in detail in Supplementary Table 6) includes Br- and Cl-initiated oxidation. Radical concentrations for Hg redox chemistry are from ref. ^57^ with a diurnal cycle based on solar zenith angle imposed on top of monthly averages. Photolysis of HgBr-X species is calculated using the GEOS-Chem implementation^58^ of the Fast-JX code^59^.

### Description of the GLEMOS model

For evaluation of the new Hg chemical mechanisms under the atmospheric conditions we apply the 3D multi-scale chemical transport model GLEMOS (Global EMEP Multi-media Modelling System). The model simulates atmospheric transport, chemical transformations and deposition of Hg species^33,34,60^. In this study the model grid has a horizontal resolution 3° × 3° and covers troposphere and lower stratosphere up to 10 hPa (ca. 30 km) with 20 irregular terrain-following sigma layers. The atmospheric transport of the tracers is driven by meteorological fields generated by the Weather Research and Forecast modelling system (WRF)^61^ fed by the operational analysis data from the European Centre for Medium-Range Weather Forecasts (ECMWF) (*ECMWF*, 2018)^62^. In the current version the model transports Hg(0) and four Hg(II) species (HgBr_2_, HgBrOH, HgBrOOH, HgBrNO_2_) as separate species. Gas-particle partitioning of Hg(II) is parameterized following ref. ^56^. A two-step mechanism of Hg(0) oxidation by Br in gas phase is included (ref. ^63^):

1 $${\mathrm H}{\mathrm g}\left(0\right)+{\mathrm X}+{\mathrm M} \to {\mathrm H} {\mathrm g}^{\mathrm I}{\mathrm X} + {\mathrm M}$$

2 $${\mathrm H}{\mathrm g}^{\mathrm I}{\mathrm X} + {\mathrm M} \to {\mathrm H}{\mathrm g}\left( 0 \right) + {\mathrm X} + {\mathrm M}$$

3 $${\mathrm H}{\mathrm g} ^{\mathrm I}{\mathrm X} + {\mathrm Y} \to {\mathrm H} {\mathrm g}\left( 0 \right) + {\mathrm X} {\mathrm Y}$$

4 $${\mathrm H}{\mathrm g}^{\mathrm I}{\mathrm X} + {\mathrm Y} + {\mathrm M} \to {\mathrm H} {\mathrm g}\left( {\mathrm {II}} \right){\mathrm X}{\mathrm Y} +{\mathrm M,}$$

The full reaction scheme is listed in Supplementary Table 6. Briefly, X ≡ Br is the first-step Hg(0) oxidant, Y is the second-step Hg(I) oxidant, and M is a molecule of air. The reaction rate constants are from: ref. ^64^ for R1; ref. ^9^ for R2; ref. ^63^ for Y ≡ Br in R3; ref. ^12^ for Y ≡ Br and OH in R4; ref. ^27^ for Y ≡ HO_2_ and NO_2_ in R4. Six-hourly concentration fields of Br are archived from a GEOS-Chem simulation^65^, whereas OH, HO_2_, NO_2_, and particulate matter (PM_2.5_) fields are imported from MOZART^66^. The aqueous-phase chemistry includes oxidation^10,67,68^ of Hg(0) by dissolved O_3_, OH and Cl(I)^I^. We have included the gas-phase photoreduction of HgBr_2_, HgBrOH, HgBrOOH, *syn*-HgBrONO using the rates calculated by CAM-Chem and the aqueous-phase photoreduction in cloud droplets with the photolysis rate constant 0.15 h^−1^ estimated in this study. We perform simulations for the period 2007–2013 using anthropogenic emissions for 2010 (AMAP/UNEP, 2013)^69^. Prescribed fluxes of Hg natural and secondary emissions from soil and seawater are generated depending on Hg concentration in soil, soil temperature and solar radiation for emissions from land and proportional to the primary production of organic carbon in seawater for emissions from the ocean^60^. The first 6 years of the period are used for the model spin up to achieve the steady-state Hg concentrations in the troposphere. The model results are presented as annual averages for 2013.

### Description of rainfall Hg(II) gross reduction rate experiments

Ten rainfall events were sampled in suburban Toulouse and at the high mountaintop Pic du Midi Observatory (France) in the summer of 2017 using ultra-clean methods^70^. Rainfall samples were transferred to a 0.5 L quartz reactor and illuminated with natural sunlight outdoors (up to 8 h), or with a solar simulator indoors (up to 48 h). Filtered samples were passed through a 0.45 micro-m quartz filter membrane to remove particles, in unfiltered samples this step was left out. Total Hg concentration of selected rainfall samples was augmented 10× with a NIST 3133 standard Hg solution, and equilibrated 24 h before light exposure. During light exposure, the quartz reactor was purged with Hg-free argon gas to remove product Hg(0). Reactant Hg(II) concentrations were measured in duplicate by cold vapour atomic fluorescence spectroscopy (CV-AFS) in 5 mL aliquots recovered from the reactor at fixed time steps and acidified to 0.04 M HCl, and 0.1 M BrCl. CV-AFS analysis accuracy was evaluated by regular analysis of the NRC ORMS-6 certified (25.6 ng L^−1^) reference material with good results (24.8 ± 1.6 ng L^−1^, 1σ, *n* = 33). Five out of twelve experiments showed increasing or constant reactant Hg(II) levels during the initial 2–4 hours, followed by a gradual decreasing in the final 24 h (Supplementary Data 1). These initial observations, tentatively explained by Hg(II)-DOM interaction with the quartz reactor wall, were not included in the rate constant calculation. This simplification did not affect the main outcome of this study. For further discussion on in-cloud Hg photoreduction see Supplementary Note.

### Code availability

The code used in this study is available upon request.

## Electronic supplementary material

Description of Additional Supplementary Files

Supplementary Information

Supplementary Data 1

Supplementary Data 2

## Data Availability

The data that supports the findings of this study is available upon request.
