# Author Correction: Photoreduction of gaseous oxidized mercury changes global atmospheric mercury speciation, transport and deposition

**DOI:** 10.1038/s41467-022-28455-w

**Published:** 2022-02-10

**Authors:** Alfonso Saiz-Lopez, Sebastian P. Sitkiewicz, Daniel Roca-Sanjuán, Josep M. Oliva-Enrich, Juan Z. Dávalos, Rafael Notario, Martin Jiskra, Yang Xu, Feiyue Wang, Colin P. Thackray, Elsie M. Sunderland, Daniel J. Jacob, Oleg Travnikov, Carlos A. Cuevas, A. Ulises Acuña, Daniel Rivero, John M. C. Plane, Douglas E. Kinnison, Jeroen E. Sonke

**Affiliations:** 1 Department of Atmospheric Chemistry and Climate, Institute of Physical Chemistry Rocasolano, CSIC, Madrid, 28006 Spain; 2 Kimika Fakultatea, Euskal Herriko Unibertsitatea UPV/EHU and Donostia International Physics Center (DIPC), P.K. 1072, Euskadi, 20080 Donostia, Spain; 3 Institut de Ciencia Molecular, Universitat de Valencia, Valencia, 46071 Spain; 4 Géosciences Environnement Toulouse, CNRS/OMP/Université de Toulouse, 31400 Toulouse, France; 5 Department of Environment and Geography, Centre for Earth Observation Science, University of Manitoba, Winnipeg, MB R3T 2N2 Canada; 6 Harvard John A. Paulson School of Engineering and Applied Sciences, Harvard University, Cambridge, MA 02138 USA; 7 Meteorological Synthesizing Centre – East of EMEP, Moscow, 115419 Russia; 8 School of Chemistry, University of Leeds, Leeds, LS2 9JT UK; 9 Atmospheric Chemistry Observations and Modelling, NCAR, Boulder, CO 80301 USA

Correction to: *Nature Communications* 10.1038/s41467-018-07075-3, published online 15 November 2018.

The original version of the Supplementary Information associated with this Article included an incorrect Supplementary Data 2 file, in which wrong spectral data were supplied. The HTML has been updated to include a corrected version of Supplementary Data 2; the original incorrect version of Supplementary Data 2 can be found as Supplementary Information associated with this Correction.

## Supplementary information

Supplementary Data 2

