# Supplementary material for: Photoreduction of gaseous oxidized mercury changes global atmospheric mercury speciation, transport and deposition
Source: Nat Commun. 2018 Nov 15;9:4796. doi: 10.1038/s41467-018-07075-3 (PMC6237998; doi:10.1038/s41467-018-07075-3)
Supplement: Supplementary file 1 — Description of Additional Supplementary Files [file 41467_2018_7075_MOESM1_ESM.pdf]

Description of Additional Supplementary Files

File Name: Supplementary Data 1
Description: Summary of rainwater photoreduction experiments. Filtered/unfiltered samples are indicated by U/F. Hg concentrations in italics are not included in the kinetic rate constants analysis, due to hysteresis at the start of experiments (see Methods). Generally, photoreduction rates were 1st order with respect to total Hg concentration, except for 12 and 24 hour samples from Pic du Midi (PDM). Rainfall event 5, under natural sunlight conditions was conducted during two consecutive days.

File Name: Supplementary Data 2
Description: Computed absorption cross sections of HgCl2, HgBr2, HgBrI, HgBrOBr, HgBrOI, HgBrOCl, HgBrNO2, HgBrONO, HgBrOH, HgBrOOH and HgO.
