# Supplementary material for: Photoreduction of gaseous oxidized mercury changes global atmospheric mercury speciation, transport and deposition
Source: Nat Commun. 2018 Nov 15;9:4796. doi: 10.1038/s41467-018-07075-3 (PMC6237998; doi:10.1038/s41467-018-07075-3)
Supplement: Supplementary file 2 — Supplementary Information [file 41467_2018_7075_MOESM2_ESM.docx]

SUPPLEMENTARY INFORMATION:

**Photoreduction of gaseous oxidized mercury changes global atmospheric mercury speciation, transport and deposition**

Saiz-Lopez et al.,

**This file contains:**

1. **Supplementary Figures 1-11**
2. **Supplementary Tables 1-6**
3. **Supplementary Note: text for in-cloud Hg photoreduction**
4. ***Supplementary Information References.***
5. **Supplementary Figures**


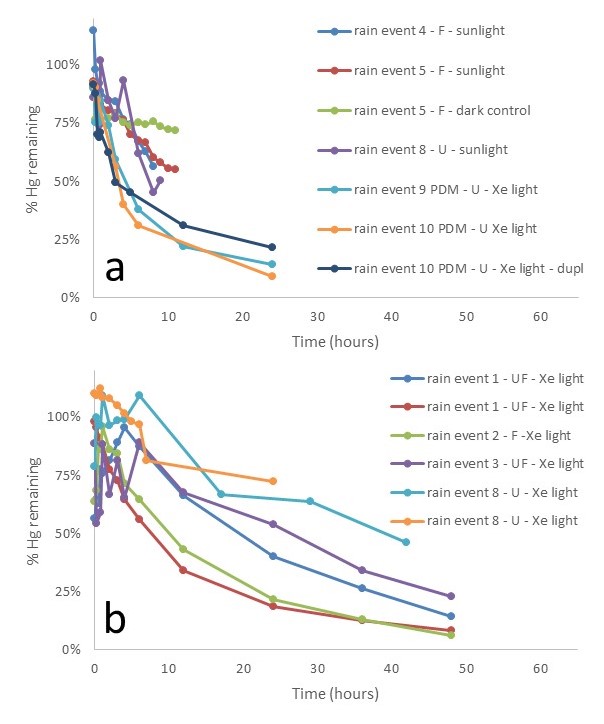


**Supplementary Figure 1**. **Experimental Hg(II) photoreduction rates** for a) short outdoors exposure of Toulouse rainfall to sunlight (events 4, 5, 8), to no light conditions (event 8 dark control), and Pic du Midi rainfall to simulated light conditions (events 9, 10), b) controlled exposure of Toulouse rainfall to simulated sunlight. (events 1, 2, 3, 8). UF and F indicate unfiltered and filtered rainfall. The combined analysis uncertainty of Hg concentration measurements was 13% (2σ level).


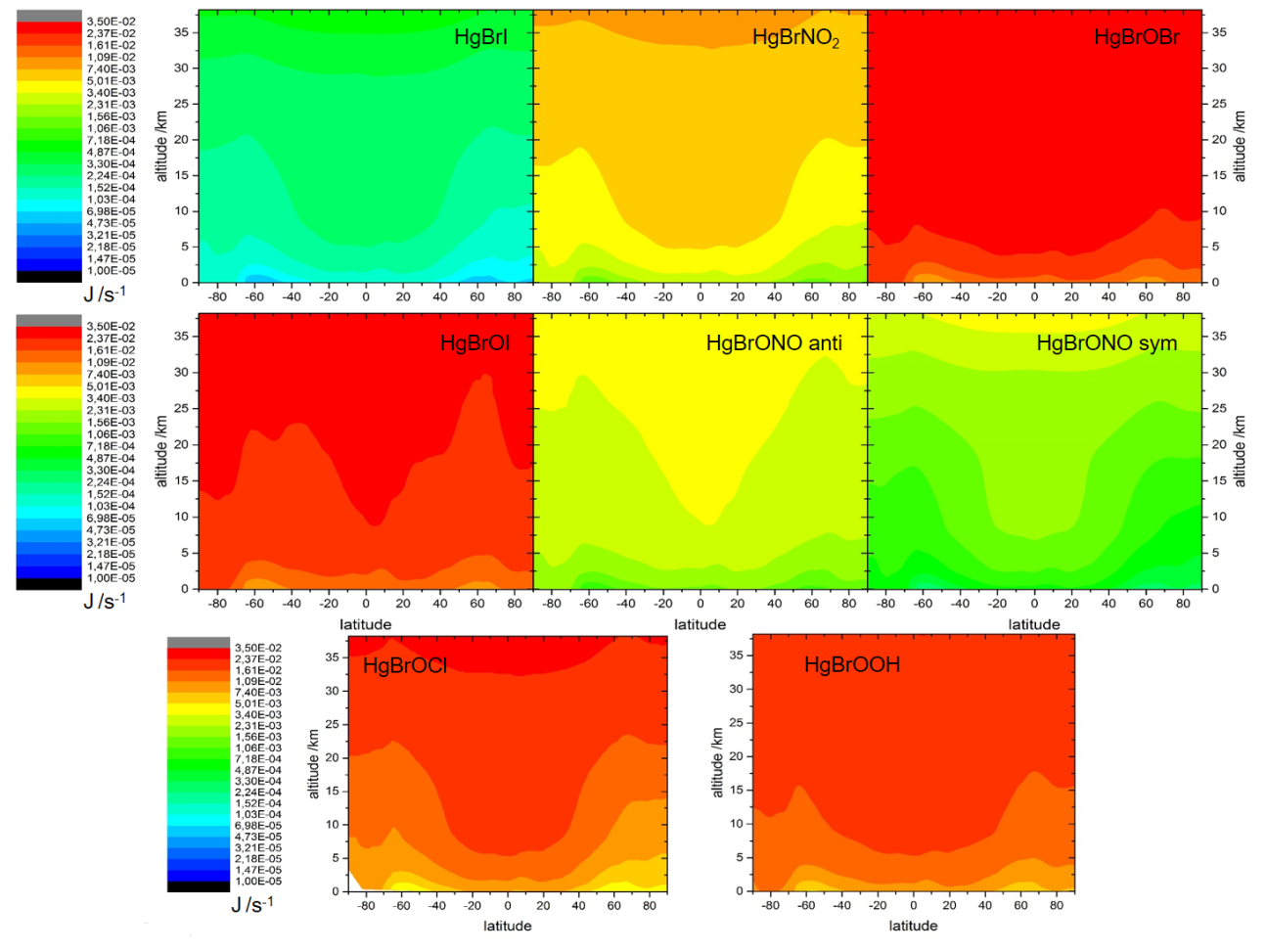


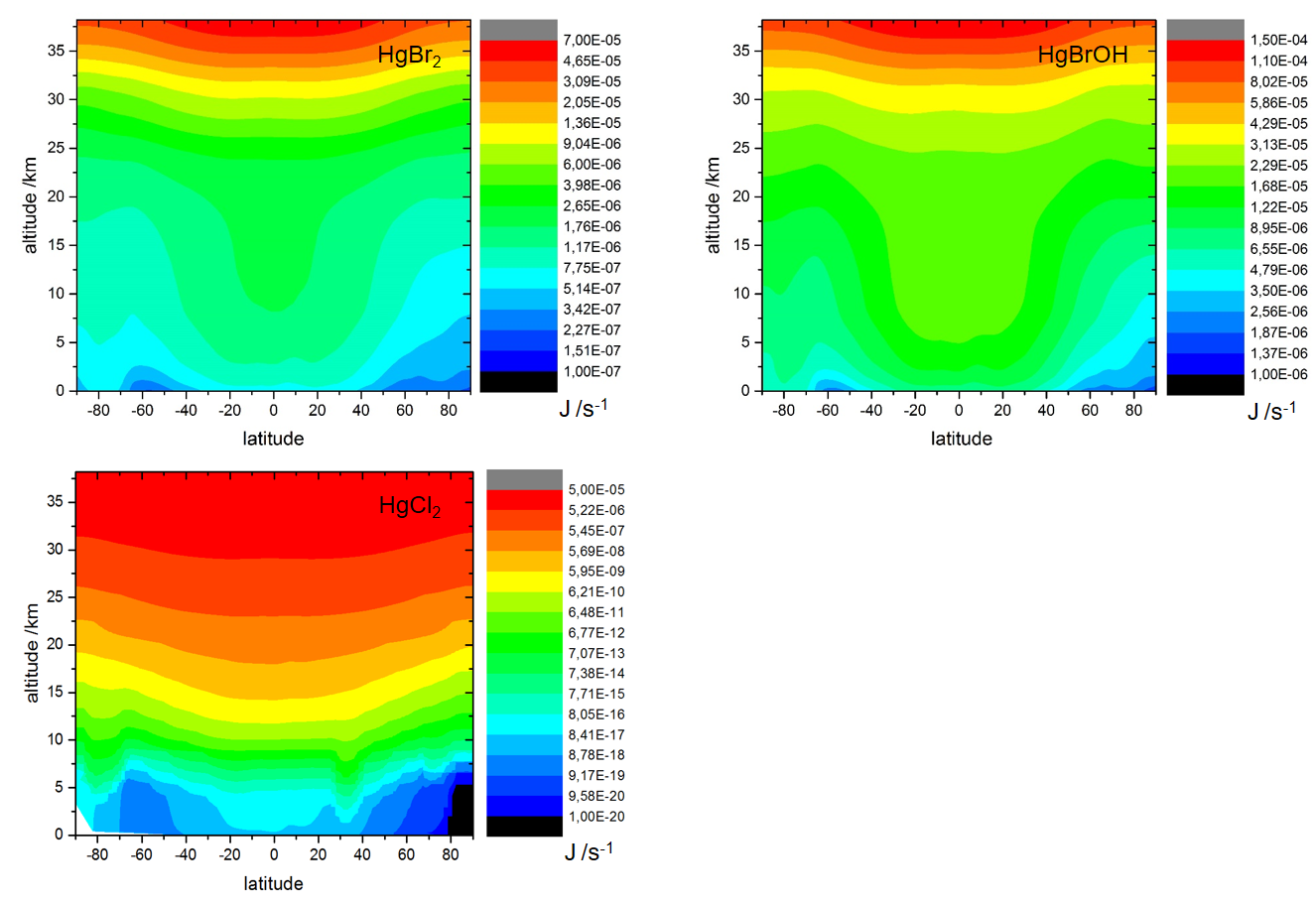


**Supplementary Figure 2**. **Photolysis rates (J, s^-1^) zonal-averaged along all longitudes for the studied Hg(II) compounds**, computed with global 3D chemistry-climate model CAM-Chem.


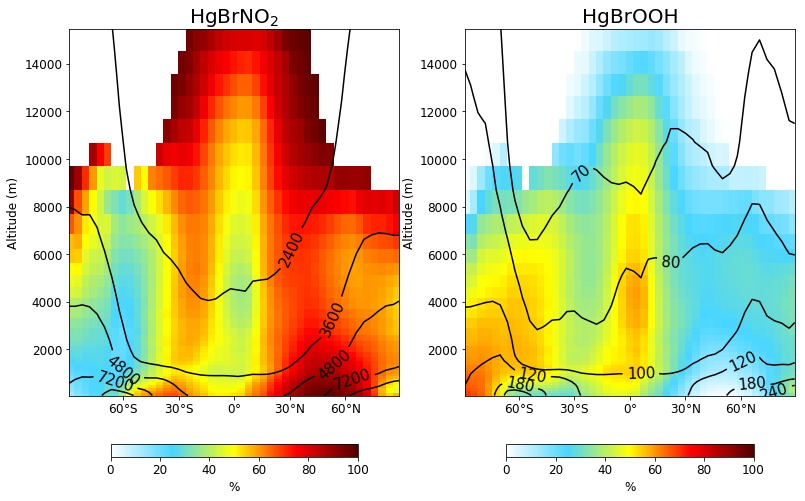


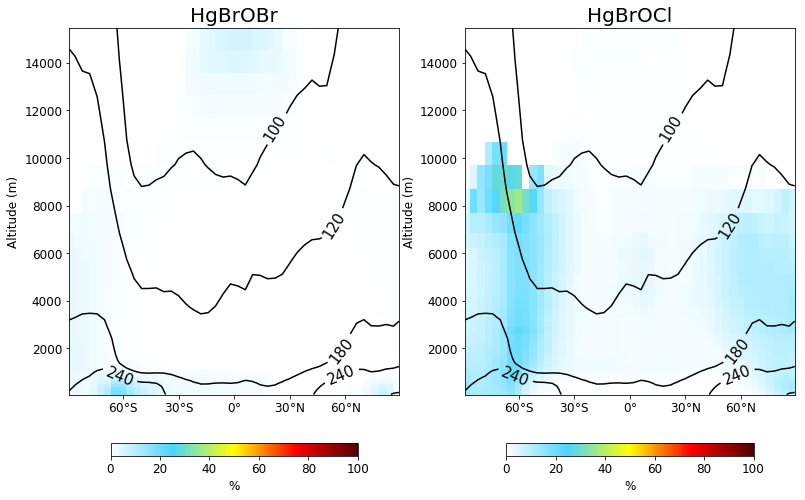


**Supplementary Figure 3**. **Fraction of HgBrX (X = BrO, ClO, *syn*-ONO, HO_2_) in colours and photolysis lifetimes (seconds) in contours modelled by GEOS-Chem**. Note that HgBrOH and HgBr_2_ are both very small fractions of HgBrX and are not shown.

| (a)  | (b)  |
| --- | --- |
| (c)  | (d)  |
|  | |

**Supplementary Figure 4. Annual zonal mean volume mixing ratios of HgBr_2_ (ppqv Hg) for different tests in GLEMOS**: (a) – Run #1; (b) – Run #2; (c) – Run #3; (d) – Run #4.

| (a)  | (b)  |
| --- | --- |
| (c)  | (d)  |
|  | |

**Supplementary Figure 5. Annual zonal mean volume mixing ratios of HgBr (ppqv Hg) for different tests in GLEMOS**: (a) – Run #1; (b) – Run #2; (c) – Run #3; (d) – Run #4.

| (a)  | (b)  |
| --- | --- |
| (c)  | (d)  |
|  | |

**Supplementary Figure 6. Annual zonal mean volume mixing ratios of Hg^0^ (ppqv) for different tests in GLEMOS**: (a) – Run #1; (b) – Run #2; (c) – Run #3; (d) – Run #4.

| (a)  | (b)  |
| --- | --- |
| (c)  | (d)  |
|  | |

**Supplementary Figure 7. Annual zonal mean volume mixing ratios of HgBrOH (ppqv Hg) for different tests in GLEMOS**: (a) – Run #1; (b) – Run #2; (c) – Run #3; (d) – Run #4.

| (a)  | (b)  |
| --- | --- |
| (c)  | (d)  |
|  | |

**Supplementary Figure 8. Annual zonal mean volume mixing ratios of HgBrOOH (ppqv Hg) for different tests in GLEMOS**: (a) – Run #1; (b) – Run #2; (c) – Run #3; (d) – Run #4.

| (a)  | (b)  |
| --- | --- |
| (c)  | (d)  |
|  | |

**Supplementary Figure 9. Annual zonal mean volume mixing ratios of *syn*-HgBrONO (ppqv Hg) for different tests in GLEMOS**: (a) – Run #1; (b) – Run #2; (c) – Run #3; (d) – Run #4.

| (a)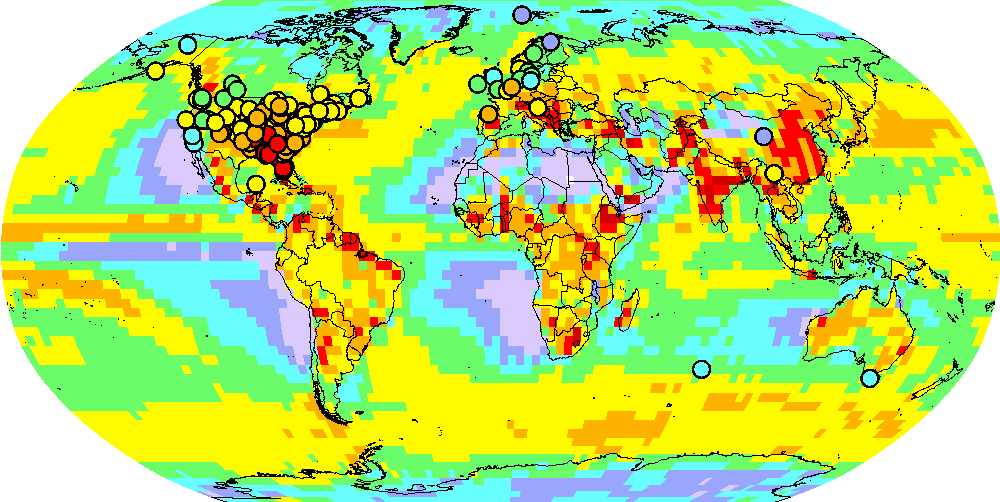 | (b)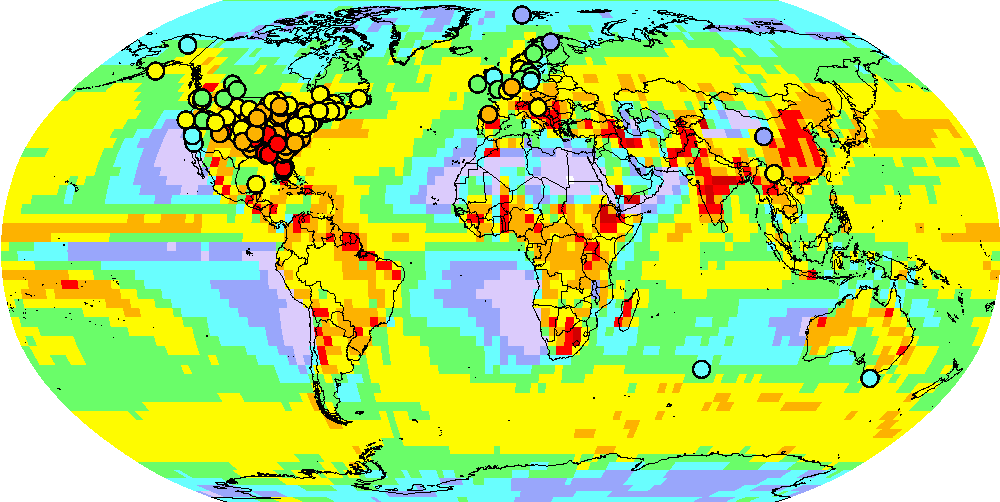 |
| --- | --- |
|  |  |
| (c)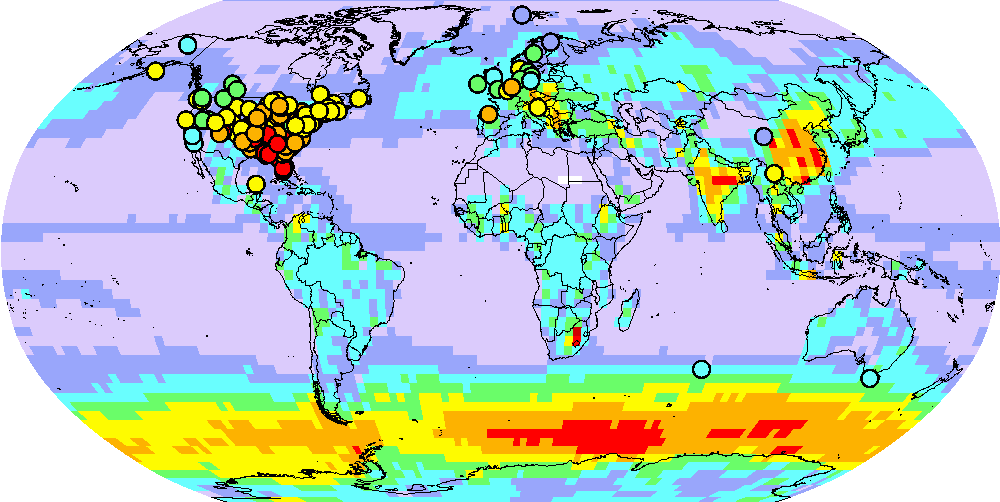 | (d)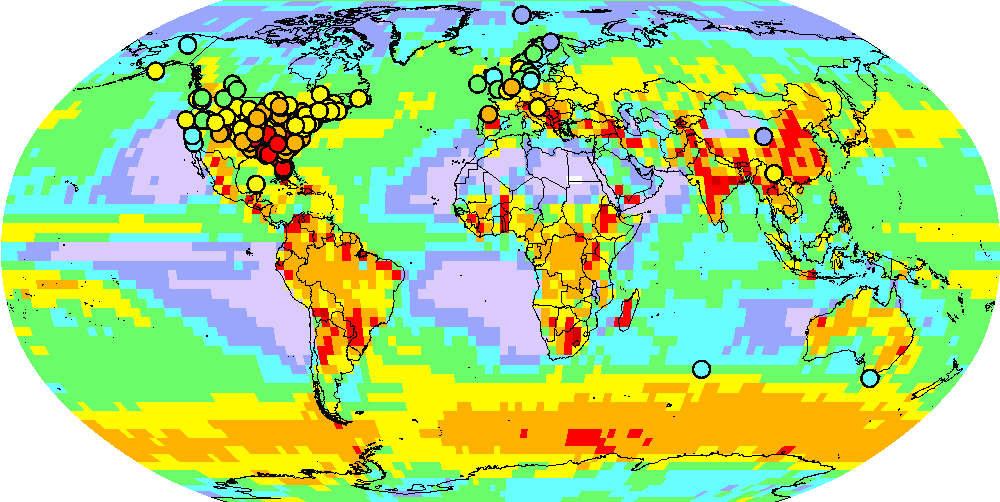 |
| 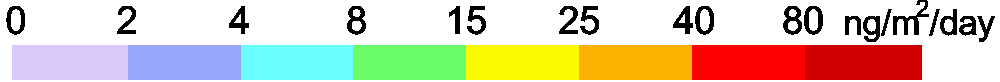 | |

**Supplementary Figure 10. Spatial distribution of Hg(II) wet deposition for different tests in GLEMOS**: (a) – Run #1; (b) – Run #2; (c) – Run #3; (d) – Run #4. Circles show observed values in the same color scale. The measurement dataset is the same as in ref Tranikov et al., 2017^1^ in the main text.


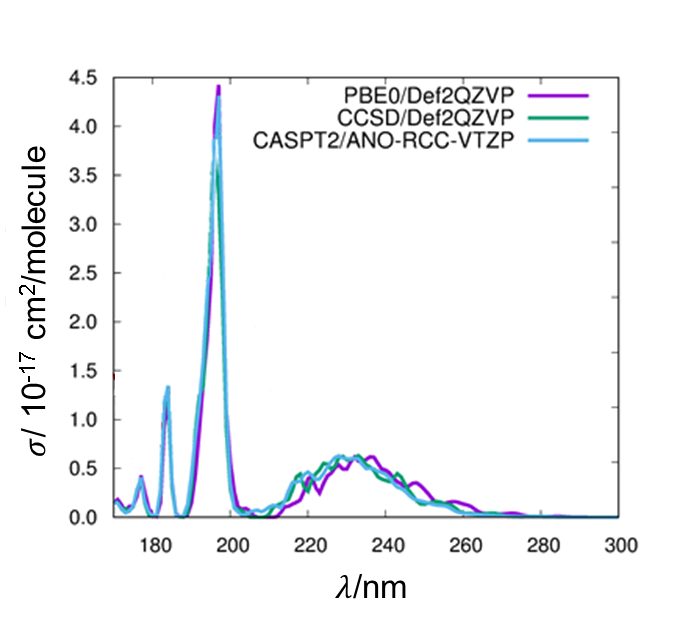


**Supplementary Figure 11.** **Calculated spectra of HgBr_2_ using different ensembles of geometries generated at different level of theory**. Electronic structure computations were done at the CASSCF/MS–CASPT2/SO–RASSI level with the ANO-RCC-VTZP basis set.

1. **Supplementary Tables**

**Supplementary Table 1**. **Summary of Hg(II) photoreduction experiments for rainfall collected in suburban Toulouse (events 1-8) and in the free troposphere of the Pic du Midi Observatory (PDM, 2877m altitude)**. UF, unfiltered; F, filtered; Xe, xenon solar simulator. Legends are referred to Supplementary Data 1.

| Experiment name | Figure legend | k_red_ (h^-1^) |
| --- | --- | --- |
|  |  |  |
| PC-R1U1XL | rain event 1 - UF - Xe light | 0.043 |
| PC-R1U2S2XL | rain event 1 - UF - Xe light | 0.071 |
| PC-R2F1S1XL | rain event 2 - F -Xe light | 0.064 |
| PC-R3U1S1XL | rain event 3 - UF - Xe light | 0.016 |
| PC-R4F1S1NL | rain event 4 - F - sunlight | 0.069 |
| PC-R5F1S1NL | rain event 5 - F - sunlight | 0.047 |
| R5F2S1DC | rain event 5 - F - dark control | 0.007 |
| PC-R8U1NL | rain event 8 - U - sunlight | 0.071 |
| PC-R8U2XL | rain event 8 - U - Xe light | 0.047 |
| PC-R8U3S1XL | rain event 8 - U - Xe light | 0.031 |
| mean |  | **0.051** |
| σ |  | 0.052 |
|  |  |  |
| PC-9 | PDM rain event 9 – U – Xe light | 0.145 |
| PC-10 | PDM rain event 10 – U – Xe light | 0.188 |
| PC-10 duplicate | PDM rain event 10 - U - Xe light | 0.136 |
| mean |  | **0.153** |
| σ |  | 0.012 |
|  |  |  |
| Selin et al., 2007^2^ | GEOS-Chem vs 7-04 | 2.9 |
| Horowitz et al., 2017^3^ | GEOS-Chem vs 9-02 | 1.0 |

**Supplementary Table 2.** **Photolysis rate (J) and annually averaged lifetime in the troposphere** $\boldsymbol{(\tau)}$ **of the Hg(II) compounds**.

| Compound | J/s^-1^ | $\boldsymbol{\tau}$ /s |
| --- | --- | --- |
| HgCl_2_ | 6.28E-10 | 1.59E+09 |
| HgBr_2_ | 9.70E-07 | 1.03E+06 |
| HgBrI | 1.67E-04 | 5.99E+03 |
| HgBrONO_*syn* | 9.60E-04 | 1.04E+03 |
| HgBrONO_*anti* | 2.31E-03 | 4.34E+02 |
| HgBrNO_2_ | 3.95E-03 | 2.53E+02 |
| HgBrOCl | 1.15E-02 | 8.70E+01 |
| HgBrOOH | 1.32E-02 | 7.57E+01 |
| HgBrOI | 1.78E-02 | 5.60E+01 |
| HgBrOBr | 2.17E-02 | 4.61E+01 |
| HgO | 5.42E-01 | 1.84E+00 |
| HgBrOH | 1.07E-05 | 9.34E+04 |

**Supplementary Table 3.** **Calculated bond dissociation energies *D*_0_ (at 0K) for BrHg−X species. All values in kJ⋅mol^-1^**.

|  | *D*_0_ |
| --- | --- |
| BrHg−Br | 305.4^4^, 303.8^5^ |
| BrHg−NO_2_ | 138.9^6^, 149.0^7^, 142.7^8^, 139.3^9^ |
| *anti*-BrHg−ONO | 156.1^6^, 160.7^7^, 151.9^8^, 150.2^9^ |
| *syn*-BrHg−ONO | 177.0^6^, 182.2^7^, 177.4^8^, 176.1^9^ |
| BrHg−OOH | 177.4^7^, 167.4^9^ |
| BrHg−OBr | 232.6^9^, 223.8^7^ |
| BrHg−OCl | 220.6^9^, 211.7^7^ |

**Supplementary Table 4.** **Statistics of the model evaluation against measurements.**

| **Run ID** | *Hg^0^ concentration* | | *Hg wet deposition* | |
| --- | --- | --- | --- | --- |
|  | *Correlation ^(a)^* | *Relative bias, % ^(b)^* | *Correlation ^(a)^* | *Relative bias, % ^(b)^* |
| Run #1 | 0.63 | -39 | 0.51 | -6.8 |
| Run #2 | 0.63 | -35 | 0.53 | -8.5 |
| Run #3 | 0.71 | 91 | 0.11 | -78 |
| Run #4 | 0.68 | 18 | 0.52 | -21 |

^(a)^ Pearson’s correlation coefficient:

$R_{corr}=\frac{\sum_{i} (M_{i}-\overline{M})(O_{i}-\overline{O})}{\sqrt{\sum_{i} (M_{i}-\overline{M})^{2}\sum_{i} (O_{i}-\overline{O})^{2}}}$;

^(b)^ Relative bias:

**$RBIAS=\frac{\overline{M}-\overline{O}}{\overline{O}}100\%$.**

**Supplementary Table 5.** **Summary of the computational details used in the quantum-chemical calculation of spectra and cross sections of mercury compounds**: geometry optimization and vibrational analysis, computation of the excited states, symmetry constraints, active space used in the CASSCF/CASPT2 calculations, number of the sampled geometries (N_p_), number of states for each irreducible representation including ground state (N_fs_), phenomenological broadening δ.

| System | HgCl_2_, HgBr_2_, HgBrI | | ^1^Σ HgO, ^3^Π HgO | | *syn*-, *anti*-HgBrONO, HgBrNO_2_ | | HgBrOBr, HgBrOI | | HgBrOH, HgBrOOH | |
| --- | --- | --- | --- | --- | --- | --- | --- | --- | --- | --- |
| Geom. opt. and vibr. analysis | Method: PBE0  Basis set: Def2QZVP | | | | | | | | | |
| Comp. of the excited states | Method: SOC-DKH3-MSCASPT2  Basis set: ANO-RCC-VTZP | | | | | | | | | |
| Symmetry | C_s_ | | C_2v_ | | C_1_ | | C_1_ | | C_1_ | |
| Active space | (12,10) | | (16,12) | | (16,12) | | (16,12) | | HgBrOH: (12,11)  HgBrOOH: (16,12) | |
| N_p_ | 100 | | 100 | | 200 | | 150 | | HgBrOH: 150  HgBrOOH: 200 | |
| N_fs_ (per Irrep) | ^1^A′ : 10  ^1^A″: 10 | ^3^A′ : 10  ^3^A″: 10 | ^1^A_1_: 8  ^1^A_2_: 8 ^1^B_1_: 8  ^1^B_2_: 8 | ^3^A_1_: 8  ^3^A_2_: 8 ^3^B_1_: 8  ^3^B_2_: 8 | ^1^A: 25 | ^3^A : 25 | ^1^A: 25 | ^3^A : 25 | ^1^A: 20 | ^3^A : 20 |
| δ [eV]^7^ | 0.05 | | | | | | | | | |

**Supplementary Table 6**. **Chemical schemes used in the GLEMOS and GEOS-Chem models**.

GLEMOS:

| **N** | **Reaction** | **Rate, molecule cm^-3^ s^-1^** | **Reference** |
| --- | --- | --- | --- |
| R1 | $Hg^{0}+Br+M\to HgBr+M$ | 1.5 × 10^-32^ (T/298)^-1.86^ [Hg^0^][Br][M] | Donohoue et al. (2006)^10^ |
| R2 | $HgBr+M\to Hg^{0}+Br+M$ | 1.6 × 10^-9^ exp(-7801/T) [HgBr][M] | Dibble et al. (2012)^9^ |
| R3 | $HgBr+Br\to Hg^{0}+Br_{2}$ | 3.9 × 10^-11^ [HgBr][Br] | Balabanov et al. (2005)^11^ |
| R4 |  | 2.5 × 10^-10^ (T/298)^-0.57^ [HgBr][Br] | Goodsite et al. (2004)^12^ |
| R5 |  | 2.5 × 10^-10^ (T/298)^-0.57^ [HgBr][OH] | Goodsite et al. (2004)^12^ |
| R6 |  | k_NO2_([M],T)[HgBr][NO_2_] | Jiao and Dibble (2017)^8^ |
| R7 |  | k_HO2_([M],T)[HgBr][HO_2_] | Jiao and Dibble (2017)^8^ |
| R8 | ,  *Y = Br, OH, NO_2_, HO_2_* | k_photo_([M],T)[HgBrY] | This study |
| R9 | ,  *Y = Br, OH, NO_2_, HO_2_* | k_photo_([M],T)[HgBrY] | This study |
| R10 | $Hg^{II}(aq)+h\nu\to Hg^{0}(aq)$ | 4.25× 10^-5^ **s^-1^** | This study |

GEOS-Chem:

|  | Reaction | Rate expression | Ref |
| --- | --- | --- | --- |
| R1 | ${Hg}^{0}+Br+M \to HgBr+M$ | 1.46 × 10^-32^ (T/298)^-1.86^[Hg^0^][Br][M] | (Donohoue et al., 2006)^10^ |
| R2 | $HgBr+M \to{Hg}^{0}+Br+M$ | 1.6 × 10^-9^ (T/298)^-1.86^ exp(-7801/T)[HgBr][M] | (Dibble et al., 2012) ^9^ |
| R3 | $HgBr+Br \to{Hg}^{0}+ {Br}_{2}$ | 3.9 × 10^-11^ [HgBr][Br] | (Balabanov et al., 2005)^11^ |
| R4 | $HgBr+Br \underset{\to}{M} HgBr$ | 3.0 × 10^-11^ [HgBr][Br] | (Balabanov et al., 2005)^11^ |
| R5 | $HgBr+{NO}_{2} \underset{\to}{M} {HgBrNO}_{2}$ | $k_{{NO}_{2}}$([M],T)[HgBr][NO_2_] | (Jiao & Dibble 2017)^8^ |
| R6 | $HgBr+Y \underset{\to}{M} HgBrY$  $Y= {HO}_{2},ClO, BrO, OH, {Br}_{2}$ | $k_{{HO}_{2}}$([M],T)[HgBr][Y] | (Jiao & Dibble 2017)^8^ |
| R7 | ${Hg}^{0}+Cl+M \to HgCl+M$ | 2.2 × 10^-22^ exp(680(1/T – 1/298))[Hg^0^][Cl][M] | (Donohoue et al., 2005)^13^ |
| R8 | $HgCl+Cl \to{Hg}^{0}+ {Cl}_{2}$ | 1.20 × 10^-11^exp(-5942/T)[HgCl][Cl] | (Wilcox 2009)^14^ |
| R9 | $HgCl+Br \underset{\to}{M} HgBrCl$ | 3.0 × 10^-11^ [HgCl][Br] | (Balabanov et al., 2005)^11^ |
| R10 | $HgCl+ {NO}_{2}\underset{\to}{M} {HgClNO}_{2}$ | $k_{{NO}_{2}}$([M],T)[HgBr][NO_2_] | (Jiao & Dibble 2017)^8^ |
| R11 | $HgCl+Y \underset{\to}{M} HgClY$  $Y= {HO}_{2},ClO, BrO, OH, {Br}_{2}$ | $k_{{HO}_{2}}$([M],T)[HgBr][Y] | (Jiao & Dibble 2017)^8^ |
| R12 | ${Hg}_{(aq)}^{0}+ O_{3(aq)} \to{Hg}_{(aq)}^{II}+products$ | 4.7 × 10^7^ [Hg^0^_(aq)_][O_3(aq)_] | (Munthe 1992)^15^ |
| R13 | ${Hg}_{(aq)}^{0}+{HOCl}_{(aq)}\to{Hg}_{(aq)}^{II}+{OH}_{(aq)}^{-}+{Cl}_{(aq)}^{-}$ | 2 × 10^6^ [Hg^0^_(aq)_][HOCl_(aq)_] | (Lin & Pehkonen 1998)^16^,(Wang & Pehkonen 2004)^17^ |
| R14 | ${Hg}_{(aq)}^{0}+{OH}_{(aq)}\to{Hg}_{(aq)}^{II}+products$ | 2.0 × 10^9^ [Hg^0^_(aq)_][OH_(aq)_] | (Lin & Pehkonen 1997)^18^,(Buxton et al., 1998)^19^ |
| R15 | ${Hg}_{(aq)}^{II}+h\to{Hg}_{(aq)}^{0}$ | 6.5 × 10^-2^ $j_{{NO}_{2}}$[OA][Hg(II)_(aq)_] | (Buxton et al., 1998)^19^ |
| R16 | $HgBrX+h \to HgBr+X$  $X= {NO}_{2},{HO}_{2},ClO, BrO, OH, Br$ | $j_{Y}$ [HgBrX] | This study |

1. **Supplementary Note: text for in-cloud Hg photoreduction**

The first global Hg CTM^20^ included Hg(II) reduction in the aqueous phase by sulfite (SO_3_^2-^), with k_red_ of 0.6 s^-1^ (2160 h^-1^) based on work by Munthe et al.1991^21^

HgSO_3_^0^_(aq)_ 🡪 Hg^0^_(aq)_ with d[HgSO_3_^0^]/dt = k_red_ x [HgSO_3_^0^] (Eq.1)

Hg(SO_3_)_2_^2-^_(aq)_ 🡪 Hg^0^_(aq)_ with d[Hg(SO_3_)_2_]/dt = k_red_ x [Hg(SO_3_)_2_] (Eq.2)

A later study that directly measured the reactant HgSO_3_ put into question the previously proposed reduction mechanism and rate constant, reporting k_red_ of 0.0106 s^-1^ (38.2 h^-1^) at pH=3 and regardless of Hg, SO_3_, and O_2_ concentrations (though not independent of other competing ligands).^22^ Shia et al.’s CTM^20^ also included aqueous phase reduction of Hg(II) by hydroperoxyl, HO_2_, radicals, based on Lin and Pehkonen (1998):^16^

Hg^2+^_(aq)_ + HO_2(aq)_ 🡪 Hg^0^_(aq)_ with d[Hg^2+^]/dt = k_red_ x [Hg^2+^] x [HO_2_] (Eq.3)

with k_red_ of 1.7 10^4^ M^-1^ s^-1^. This reaction, which proceeds by a Hg(I) intermediate step has been questioned by Gardfeldt and Jonsson^23^ based on the rapid oxidation of the Hg(I) form back to Hg(II). It is also important to note that the reaction rate expressions Eq.1 and 2 depend on free Hg^2+^ or HgSO_3_^0^ ion concentrations, which cannot be measured and have to be estimated based on equilibrium speciation calculations. In the 1990’s and early 2000’s it was generally assumed that inorganic ligands such as Cl- were among the dominant Hg binding ligands in atmospheric waters, or that high SO_3_ concentrations in polluted areas inhibited Hg reduction via the slower Eq. 2. The subsequent gradual acceptance of simple and complex (humics) dissolved organic ligands as dominant Hg binding ligand in natural waters, including rainfall^24^ results in low HgSO_3_, Hg(SO_3_)_2_ and even lower free Hg^2+^ ion concentrations.^25,26^ Consequently these aqueous phase Hg(II) reduction mechanisms have been gradually abandoned in Hg CTMs.

The uncertainty in atmospheric Hg(II) reduction pathways has led subsequent models to adopt a fitting approach (e.g. CTM-Hg, GEOS-Chem) to aqueous phase Hg(II) reduction.^27,2^ GEOS-Chem provides the most explicit parameterization that is well suited for comparison to our experimental results. Selin et al. (2007)^2^ optimized in-cloud Hg(II) photoreduction in the GEOS-Chem CTM as k_red_ = 8 10^-10^ [OH] (s^-1^) where [OH] is the gas phase concentration in units of molecules cm^-3^. [OH] serves as a proxy for the actinic flux in the VIS region. For a typical [OH] of 10^6^ , k_red_ is 8 10^-4^ s^-1^ (2.9 h^-1^) which resulted in a mean lifetime of 20 min for dissolved Hg(II) in cloud in the model. Note that the associated rate expression, d[Hg(II)]/dt=k_red_ x [Hg(II)] is expressed relative to total divalent Hg(II) concentration in cloud water. Horowitz et al. (2017),^3^ using the most recent two-step fast Hg^0^ oxidation scheme in GEOS-Chem, extended atmospheric Hg(II) reduction to aqueous-phase Hg(II)–organic complexes in both aqueous aerosols and clouds. k_red_ (s^-1^) is parameterized as α*j*NO_2_[OA], were α is a scaling factor, *j*NO_2_ the NO_2_ photolysis frequency, a proxy for the UV actinic flux, [OA] the local concentration of organic aerosol. If we take generic values of *j*NO2 = 10^-2^ s^-1^ and maximum [OA] = 1 ug m^-3^ (STP), we obtain a maximum rate constant of 1 h^-1^.

Following an experimental study on aqueous Hg(II) reduction by dicarboxylic acids (Hg^2+^_aq_ + DCA_aq_ + hv → Hg^0^_aq_) by Si and Ariya (2008),^28^ Bash et al. (2014)^24^ recently incorporated this reaction into the CMAQ model using a rate of 1.2 × 10^4^ M^−1^·s^−1^ (the highest rate observed for oxalic acid). Global oxalic acid concentrations in rainfall and cloud water are on the order of 5 µeq/L (2.5 µM), yielding an approximate maximum k_red_ of 1.2 10^4^ x 2.5 10^-6^ = 0.03 s^-1^ (108 h^-1^) for the reaction rate expression d[Hg^2+^]/dt=k_red_[Hg^2+^]. As argued above, free Hg^2+^ (likely < 10^-15^ mol/L) is only a very small fraction of the total Hg(II) concentration.

Therefore, a conditional rate constant relative to total Hg(II) will be orders of magnitude slower.

Supplementary Data 1 summarizes the rainfall events, rainfall pH and incubation conditions (filtration, UVA, UVA, VIS) used in this study. pH was measured using an Orion pH electrode, calibrated against NIST traceable standards of pH 4 and 7. UVA, UVB, VIS intensity of outdoors sunlight and of the solar simulator (xenon lamp) were measured with a Solar Light PMA2200 Radiometer with specific UVB (PMA2106), UVA (PMA2110) and Visible (PMA2130) detectors. Additional details can be found in the Methods section. Photoreduction rates were generally 1^st^ order with respect to total Hg concentrations during the first 12 hours. Duplicate incubations of rainfall samples 1, 8 and 10 indicate a mean RSD of 29% on k_red_.

1. **Supplementary Information References**

1. Travnikov O*, et al.* Multi-model study of mercury dispersion in the atmosphere: atmospheric processes and model evaluation. *Atmospheric Chem Phys* **17**, 5271 (2017).

2. Selin NE*, et al.* Chemical cycling and deposition of atmospheric mercury: Global constraints from observations. *J Geophys Res Atmos* **112**, (2007).

3. Horowitz HM*, et al.* A new mechanism for atmospheric mercury redox chemistry: Implications for the global mercury budget. *Atmos Chem Phys* **17**, 6353–6371 (2017).

4. Balabanov NB, Peterson KA. Mercury and reactive halogens: the thermochemistry of Hg+{Cl2, Br2, BrCl, ClO, and BrO}. *J Phys Chem A* **107**, 7465-7470 (2003).

5. Shepler BC, Balabanov NB, Peterson KA. Hg+ Br→ Hg Br recombination and collision-induced dissociation dynamics. *J Chem Phys* **127**, 164304 (2007).

6. Dibble TS, Zelie MJ, Jiao Y. Quantum Chemistry Guide to PTRMS Studies of As-Yet Undetected Products of the Bromine-Atom Initiated Oxidation of Gaseous Elemental Mercury. *J Phys Chem A* **118**, 7847-7854 (2014).

7. Jiao Y, Dibble TS. Quality Structures, Vibrational Frequencies, and Thermochemistry of the Products of Reaction of BrHg• with NO2, HO2, ClO, BrO, and IO. *J Phys Chem A* **119**, 10502-10510 (2015).

8. Jiao Y, Dibble TS. First kinetic study of the atmospherically important reactions BrHg˙+ NO2 and BrHg˙+ HOO. *Phys Chem Chem Phys* **19**, 1826-1838 (2017).

9. Dibble TS, Zelie MJ, Mao H. Thermodynamics of reactions of ClHg and BrHg radicals with atmospherically abundant free radicals. *Atmos Chem Phys* **12**, 10271-10279 (2012).

10. Donohoue DL, Bauer D, Cossairt B, Hynes AJ. Temperature and pressure dependent rate coefficients for the reaction of Hg with Br and the reaction of Br with Br: A pulsed laser photolysis-pulsed laser induced fluorescence study. *J Phys Chem A* **110**, 6623-6632 (2006).

11. Balabanov NB, Shepler BC, Peterson KA. Accurate global potential energy surface and reaction dynamics for the ground state of HgBr2. *J Phys Chem A* **109**, 8765-8773 (2005).

12. Goodsite ME, Plane J, Skov H. A theoretical study of the oxidation of Hg0 to HgBr2 in the troposphere. *Environ Sci Technol* **38**, 1772-1776 (2004).

13. Donohoue DL, Bauer D, Hynes AJ. Temperature and Pressure Dependent Rate Coefficients for the Reaction of Hg with Cl and the Reaction of Cl with Cl:  A Pulsed Laser Photolysis−Pulsed Laser Induced Fluorescence Study. *The Journal of Physical Chemistry A* **109**, 7732-7741 (2005).

14. Wilcox J. A Kinetic Investigation of High-Temperature Mercury Oxidation by Chlorine. *The Journal of Physical Chemistry A* **113**, 6633-6639 (2009).

15. Munthe J. The aqueous oxidation of elemental mercury by ozone. *Atmospheric Environ* **26**, 1461-1468 (1992).

16. Lin C-J, Pehkonen SO. Two-phase model of mercury chemistry in the atmosphere. *Atmospheric Environment* **32**, 2543-2558 (1998).

17. Wang Z, Pehkonen SO. Oxidation of elemental mercury by aqueous bromine: atmospheric implications. *Atmospheric Environment* **38**, 3675-3688 (2004).

18. Lin C-j, Pehkonen SO. Aqueous free radical chemistry of mercury in the presence of iron oxides and ambient aerosol. *Atmospheric Environment* **31**, 4125-4137 (1997).

19. Buxton GV, Greenstock CL, Helman WP, Ross AB. Critical Review of rate constants for reactions of hydrated electrons, hydrogen atoms and hydroxyl radicals (⋅OH/⋅O− in Aqueous Solution. *Journal of Physical and Chemical Reference Data* **17**, 513-886 (1988).

20. Shia RL, Seigneur C, Pai P, Ko M, Sze ND. Global simulation of atmospheric mercury concentrations and deposition fluxes. *J Geophys Res Atmos* **104**, 23747-23760 (1999).

21. Munthe J, Xiao ZF, Lindqvist O. The aqueous reduction of divalent mercury by sulfite. *Water Air & Soil Pollution* **56**, 621-630 (1991).

22. Van Loon L, Mader E, Scott SL. Reduction of the Aqueous Mercuric Ion by Sulfite:  UV Spectrum of HgSO3 and Its Intramolecular Redox Reaction. *The Journal of Physical Chemistry A* **104**, 1621-1626 (2000).

23. Gårdfeldt K, Jonsson M. Is Bimolecular Reduction of Hg(II) Complexes Possible in Aqueous Systems of Environmental Importance. *The Journal of Physical Chemistry A* **107**, 4478-4482 (2003).

24. Bash J, Carlton A, Hutzell W, Bullock Jr. O. Regional Air Quality Model Application of the Aqueous-Phase Photo Reduction of Atmospheric Oxidized Mercury by Dicarboxylic Acids. *Atmosphere* **5**, 1 (2014).

25. Haitzer M, Aiken GR, Ryan JN. Binding of Mercury(II) to Dissolved Organic Matter:  The Role of the Mercury-to-DOM Concentration Ratio. *Environmental Science & Technology* **36**, 3564-3570 (2002).

26. Tipping E. Modelling the interactions of Hg(II) and methylmercury with humic substances using WHAM/Model VI. *Applied Geochemistry* **22**, 1624-1635 (2007).

27. Seigneur C, Vijayaraghavan K, Lohman K. Atmospheric mercury chemistry: Sensitivity of global model simulations to chemical reactions. *J Geophys Res Atmos* **111**, D22306 (2006).

28. Si L, Ariya PA. Reduction of Oxidized Mercury Species by Dicarboxylic Acids (C2−C4): Kinetic and Product Studies. *Environmental Science & Technology* **42**, 5150-5155 (2008).
